# Impacts of continuous cropping on the rhizospheric and endospheric microbial communities and root exudates of *Astragalus mongholicus*

**DOI:** 10.1186/s12870-024-05024-5

**Published:** 2024-04-26

**Authors:** Qin Zhou, Yun Wang, Liang Yue, Ailing Ye, Xiaofan Xie, Meilan Zhang, Yuan Tian, Yang Liu, Andéole Niyongabo Turatsinze, Uwaremwe Constantine, Xia Zhao, Yubao Zhang, Ruoyu Wang

**Affiliations:** 1 Gansu Gaolan Field Scientific Observation and Research Station for Agricultural Ecosystem, Northwest Institute of Eco-Environment and Resources, Chinese Academy of Sciences, Lanzhou, 730000 Gansu China; 2 Key Laboratory of Stress Physiology and Ecology in Cold and Arid Regions of Gansu Province, Lanzhou, 730000 China; 3 General Station of Gansu Cultivated Land Quality Construction and Protection, Lanzhou, 730000 China

**Keywords:** *A. mongholicus*, Endogenous fungi, Rhizosphere metabolomics, Root exudates

## Abstract

*Astragalus mongholicus* is a medicinal plant that is known to decrease in quality in response to continuous cropping. However, the differences in the root-associated microbiome and root exudates in the rhizosphere soil that may lead to these decreases are barely under studies. We investigated the plant biomass production, root-associated microbiota, and root exudates of *A. mongholicus* grown in two different fields: virgin soil (Field I) and in a long-term continuous cropping field (Field II). Virgin soil is soil that has never been cultivated for *A. mongholicus*. Plant physiological measurements showed reduced fresh and dry weight of *A. mongholicus* under continuous cropping conditions (i.e. Field II). High-throughput sequencing of the fungal and bacterial communities revealed differences in fungal diversity between samples from the two fields, including enrichment of potentially pathogenic fungi in the roots of *A. mongholicus* grown in Field II. Metabolomic analysis yielded 20 compounds in *A. mongholicus* root exudates that differed in relative abundance between rhizosphere samples from the two fields. Four of these metabolites (2-aminophenol, quinic acid, tartaric acid, and maleamate) inhibited the growth of *A. mongholicus*, the soil-borne pathogen *Fusarium oxysporum*, or both. This comprehensive analysis enhances our understanding of the *A. mongholicus* microbiome, root exudates, and interactions between the two in response to continuous cropping. These results offer new information for future design of effective, economical approaches to achieving food security.

**Supplementary Information:**

The online version contains supplementary material available at 10.1186/s12870-024-05024-5.

## Background

Continuous cropping, also known as monocropping, can lead to poor crop growth and even yield failure with long-term application [1, 2]. Use of this method is therefore a major factor that limits practical crop production, and the seriousness, regularity, and duration of continuous cropping challenges are expected to increase across the world [3]. Problems associated with continuous cropping have been widely documented in many crops, such as rice [4], maize [5], and cotton [6]. However, this approach is especially damaging to medicinal plants, including *Panax ginseng* [7], *Panax notoginseng* [8], and *Rehmannia glutinosa* [9, 10]. Studies addressing the effects of continuous cropping have found that it has strong negative impacts on both plant and soil systems. These impacts include morphological, anatomical, physiological, and biochemical alterations in plants [11], altered soil microbial community structure [12], and accumulated allelopathic autotoxicity in the soil [13].

Endophytic microbes are plant-associated fungi and bacteria that inhabit all known plant compartments [14]. Many have beneficial effects, such as promoting plant growth, root elongation, seed germination, and yield [15–18]. However, continuous cropping can alter the plant-associated microbial community structure, abolishing benefits to the plant or even causing disease [8, 19]. For example, a continuous cropping experiment involving the wild tobacco plant (Nicotiana attenuata) showed that seven years of continuous cultivation led to reduced endophytic microbial abundance and increased levels of disease caused by fungal pathogens in the genera *Fusarium* and *Alternaria* [20–22]. In *Atractylodes macrocephala*, continuous cropping significantly decreases the diversity of fungal communities within the roots, stems, leaves, and tubes [23]. Although several studies such as these have focused on changes in the rhizosphere microbiome caused by continuous cropping, the precise relationships between plant roots and endophytes in the context of continuous cropping remain unclear.

Root exudates are large quantities of metabolites released from living plant root hairs or fibrous root systems into the surrounding growth substrate [24, 25]. Microbial life in the rhizosphere is nourished by plant-derived growth substrates in root exudates. Some of these substances are plant specialized metabolites, such as flavonoids, terpenoids, alkaloids, coumarins, and benzoxazinoids, which act as messengers and play distinct roles in shaping the composition and function of the rhizosphere microbiome [26–31]. In return, the rhizosphere microbiota can directly enhance plant performance by providing nutrients, suppression pathogen outbreaks, or modulating abiotic stress tolerance [32–34]. However, the details of metabolic plant–microbe interactions in continuous cropping conditions are largely unknown.

Roots also produce a class of specialized metabolites, allelochemicals that have direct or indirect adverse effects on host plant growth [35]. For example, the phenolic acids in American ginseng (*Panax quinquefolius, L.*) root exudates inhibit radicle growth in a concentration-dependent manner [36]. Importantly, allelochemicals production is associated with continuous cropping [37, 38]. For instance, Huang et al. [38] identified p-hydroxybenzoic acid as an allelochemical secreted by cucumber (*Cucumis sativus L.*) roots under continuous cropping conditions. Such prior results suggest that allelochemicals may be responsible for continuous cropping obstacles in some systems.

*Astragalus membranaceus* Bge. var. *mongholicus* (Bge.) Hsiao (hereafter referred to as *A. mongholicus*) is a member of the family *Leguminosae* and a prized material in Chinese herbal medicine [39, 40]. Large-scale commercial production of this perennial plant takes place in Longxi County, Gansu Province [41–43]. However, long-term continuous cropping in that region has compromised *A. mongholicus* growth, causing deterioration in root quality and increased incidence of root rot disease. This problem has intensified over several years, and *A. mongholicus* now fails to grow in many fields in Gansu. However, the primary factors in this system that underlie the adverse reactions to continuous cropping remain unclear.

To identify factors contributing to obstacles in continuous *A. mongholicus* cropping, we here examined differences in the associated microbiota and the root exudates produced by *A. mongholicus* grown in virgin soil or under continuous cropping conditions. The effects of root exudate-derived specialized metabolites on the growth of soil-borne phytopathogens and *A. mongholicus* were also investigated. We hypothesize that continuous cropping enriched phytopathogens in the soil; plants secrete large volumes of root exudates to defend those pathogens by themselves, but the exudates contain counterproductive autotoxic metabolites as well. The results of this study enhance our understanding of the changes induced in root endospheric microbial communities and root exudates by long-term (≥ 20 years) continuous cultivation of *A. mongholicus*. These data could be used to design an effective control strategy to suppress the adverse effects of continuous *A. mongholicus* cropping in the northwest region of China.

## Results

### Effects of continuous cropping on *A. mongholicus* growth

**Fig. 1:**
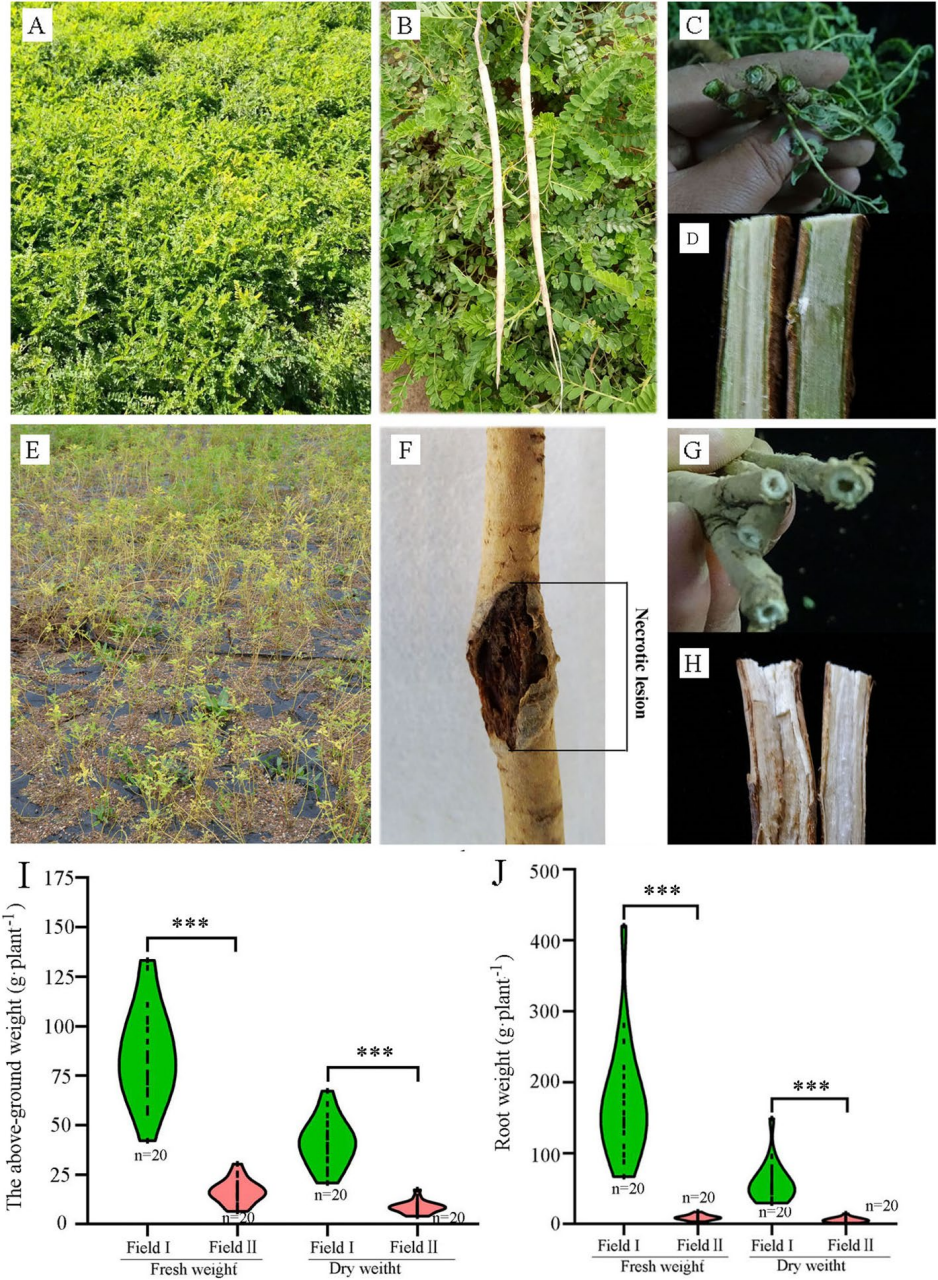
The phenotypes and biomass of *A. mongholicus* plants grown in Field I and Field II. The above- (leaves and stems) (**A**) and below-ground (roots) (**B**) parts of *A. mongholicus* in Field I, and its stem’s transverse section (**C**) and longitudinal section (**D**). The above- (**E**) and under-ground (**F**) parts of *A. mongholicus* in Field II, and its stem’s transverse section (**G**) and longitudinal section (**H**). Violin plots of the above-ground fresh and dry biomass weight of *A. mongholicus* in Field I and Field II (*n* = 20) (I). Violin plots showing the fresh weight and dry weight of *A. mongholicus* roots grown in Field I and Field II (**J**). *** *p* < 0.001 (Student’s t-test)

We first assessed the morphological phenotypes of *A. mongholicus* plants grown in virgin soil (Field I) and those grown in a field that has been continuously planted with *A. mongholicus* for at least 20 years (Field II). Overall, both the roots and aerial tissues of *A. mongholicus* plants grown in Field I appeared healthy and strong (Fig. 1A, B). In contrast, *A. mongholicus* plants grown in Field II demonstrated leaf chlorosis, root necrosis, and rapid death (Fig. 1E, F). Furthermore, *A. mongholicus* stem cross-section showed that plants grown in Field II had hollow stems (Fig. 1G), and vertical stem sections revealed white, flocculent interiors (Fig. 1H). Plants grown in Field I had normal stems (Fig. 1C, D). This indicated that continuous cropping damaged the stem tissue structure. Moreover, both the fresh and dry weight of the phyllosphere tissue (comprising the pooled leaves, stems, and branches) were significantly reduced among plants grown in Field II (by 88% and 90%, respectively, compared to those grown in Field I) (Fig. 1I). The reductions in below-ground tissue weight were slightly lower, at 78% and 79% of the fresh and dry weight, respectively (Fig. 1J). The statistical results of fresh and dry weight of phyllosphere tissue and below-ground tissue were consistent with those observed in 2019 (Fig. S1). These results confirmed that long-term continuous cropping severely compromised *A. mongholicus* growth.

## Amplicon sequencing results

**Fig. 2:**
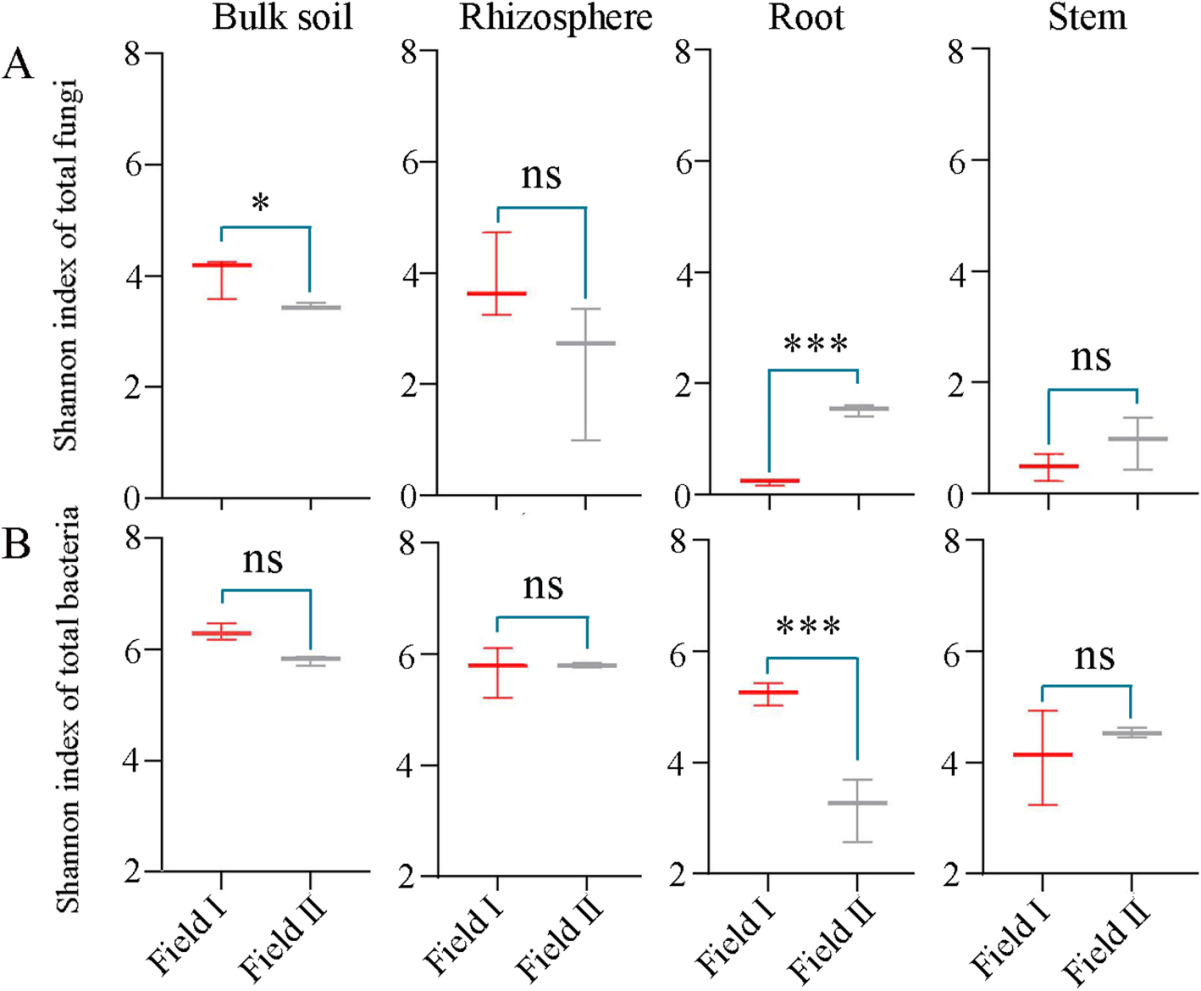
Alpha-diversity (Shannon index) for fungi (**A**) and for bacteria (**B**) present in bulk soils, rhizosphere soils, roots, and stems of *A. mongholicus* plants grown in Field I and Field II. Statistical comparisons were made used the paired Wilcoxon rank-sum test, with significance denoted by asterisks: * *p* < 0.5, *** *p* < 0.001. Values shown are the median ± SDs (*n* = 3)

We examined the fungal and bacterial communities in the rhizosphere soil, bulk soil, roots, and stems to investigate the impact of continuous cultivation on the *A. mongholicus* microbiome. The Shannon index was used to assess within-sample (α) diversity for fungi (Fig. 2A) and bacteria (Fig. 2B). Few differences between samples from Field I and Field II were statistically significant. However, there was slightly lower fungal diversity in the bulk soil collected form Field II compared to Field I (*p* < 0.05). Moreover, *A. mongholicus* roots from plants grown in Field II had significantly greater diversity in endophytic fungi compared to plants grown in Field I, but significantly lower diversity in endophytic bacteria. These results suggested that long-term continuous cropping influenced the diversity of the *A. mongholicus* root endophytic fungal and bacterial communities.

## Effects of continuous cropping on the rhizospheric and endophytic fungal community structure

**Fig. 3:**
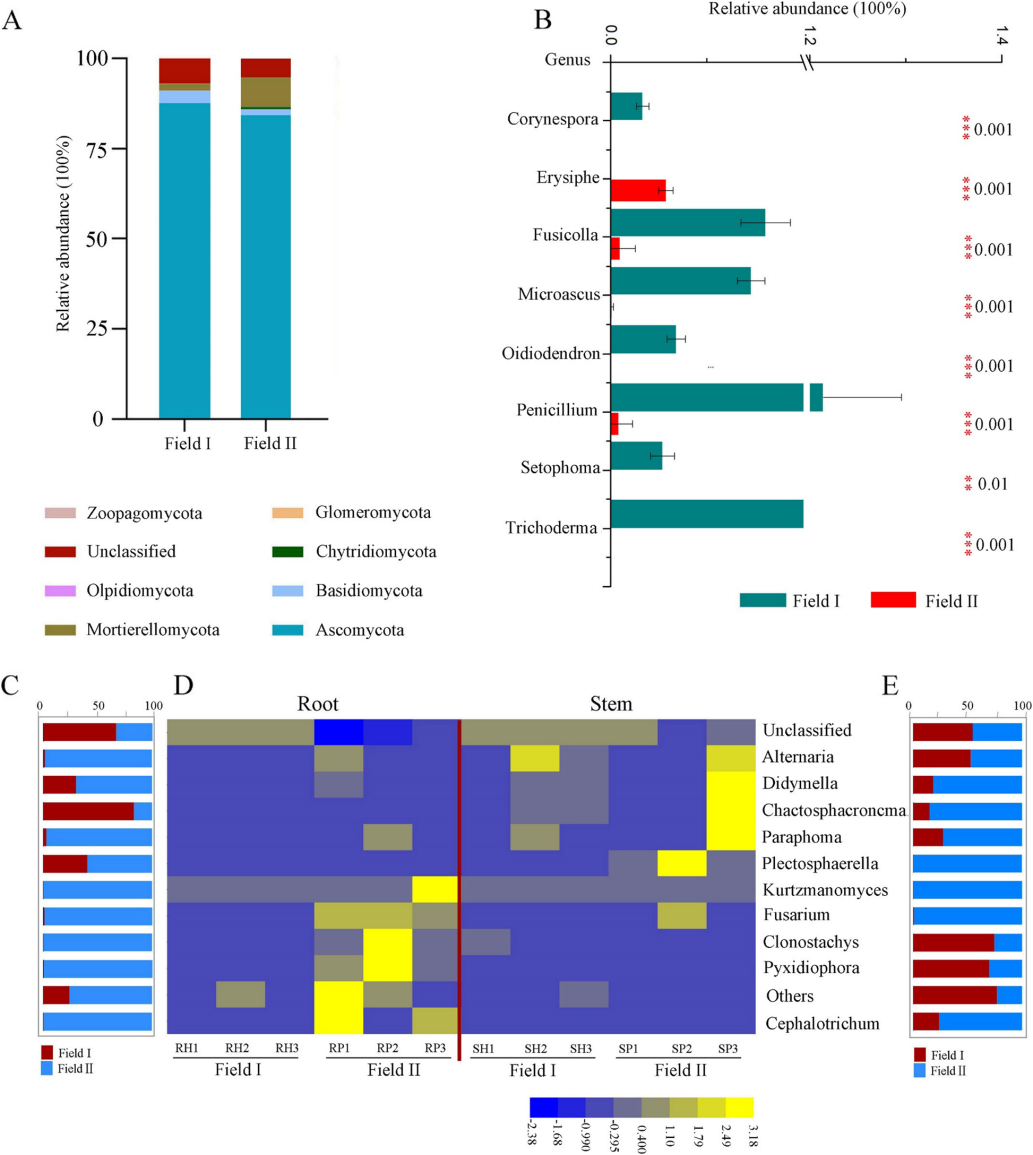
Fungal communities in the rhizosphere and endophytic compartment of *A. mongholicus* plants grown in Field I and Field II. **A** Taxonomic classification of fungal OTUs grouped at the phylum level. The two bars represent the relative abundance of fungal phyla in the rhizosphere of *A. mongholicus* grown in Field I and Field II. Unclassified represents those species not taxonomically annotated. **B** The relative abundance (%) of members of specific genera enriched in the rhizosphere soil of *A. mongholicus* grown in Field I and Field II, and likewise for of all genera presented here, was significantly different (Student’s t-test: ** *p* < 0.01, *** *p* < 0.001) between the two fields. **C** The percentage converted from the proportion of relative abundance of the top 10 endophytic fungi at the genus level in *A. mongholicus* roots grown in Field I and Field II. **D** Heatmap analysis of the relative abundance of the top 10 endophytic fungi at genus level in *A. mongholicus* roots and stems grown in Field I and Field II. **E** The percentage converted from the proportion of relative abundance of the top 10 endophytic fungi at genus level in *A. mongholicus* stems grown in Field I and Field II

Fungal phyla with relative abundance > 2% were analyzed to identify differences between the fungal communities associated with plants grown in virgin or continuously cropped soil. Ascomycota, Mortierellomycota, and Basidiomycota were the primary fungal phyla present in the rhizosphere of plants grown in both fields (Fig. 3A). However, a comparative analysis revealed significant depletion of Basidiomycota, but enrichment of Mortierellomycota, in the rhizosphere compartment of *A. mongholicus* grown in Field II compared with Field I (Fig. 3A). At the genus level, the relative abundance of *Erysiphe*, a member of the phylum Ascomycota, was significantly increased in the rhizosphere soil of *A. mongholicus* grown in Field II compared to Field I. However, levels of several other genera in the phylum Ascomycota (namely *Corynerspora*, *Fusicolla*, *Micrascus*, *Oidiodendron*, *Penicillium*, *Setophoma*, and *Trichoderma*) were significantly decreased among plants grown in Field II (Fig. 3B). Ascomycota, Mortierellomycota, and Basidiomycota were the main phyla present in the bulk soil fungal communities in both Field I and Field II, consistent with the corresponding rhizosphere samples (Fig. S3A). At the genus level, the relative abundances of some members of the phyla Ascomycota (*Brunneochlamydosporium* and *Dactylonectria*), and Basidiomycota (*Filobasidium*) were significantly higher in bulk soil from Field II than Field I, although levels of other Ascomycota (*Chaetomium*) and Basidiomycota (*Clitopilus* and *Conocybe*) were lower (Fig. S3B). Overall, these results demonstrated a lack of consistency in the fungal taxa that were differentially abundant between fields in the bulk soil and the rhizosphere.

The root and stem endophytic fungi among plants grown in either field belonged to only two phyla, Ascomycota and Basidiomycota. The phylum Ascomycota was significantly enriched in the root and stem compartments of *A. mongholicus* grown in Field II compared to those grown in Field I (Fig. S4). At the genus level, the relative abundance of the *Fusarium* (Ascomycota) was significantly increased in the roots of *A. mongholicus* grown in Field II compared to Field I (false discovery rate [FDR]-adjusted p < 0.05, Mann–Whitney* U* test) (Fig. 3C, D). Additionally, the abundances of some genera belonging to the phyla Ascomycota (namely *Cephalotrichum*, *Pyxidiophora* and *Clonostachys*) and Basidiomycota (*Kurtzmanomyces*) were higher among the roots of *A. mongholicus* grown in Field II (proportion of relative abundance over 98%) than Field I (proportion below 2%) (Fig. 3C, D). Furthermore, we found that *Alternaria* and *Paraphoma* (Ascomycota) were more abundant among *A. mongholicus* roots from plants grown in Field II (> 90% and < 98%) than Field I (> 2% and < 10%, respectively) (Fig. 3C, D). In the stems, only one genus, *Plectosphaerella* (Ascomycota), was more abundant among plants grown in Field II than in Field I (Fig. 3D). The genus *Fusarium* (Ascomycota) was also more prevalent in the stems of *A. mongholicus* grown in Field II (> 85%) than Field I (< 15%) (Fig. 3D, E). In total, these results indicated that continuous cropping altered the fungal microbial communities both within and surrounding *A. mongholicus*, including increasing the abundance of potentially pathogen genera such as *Alternaria* and *Fusarium*.

## Effects of continuous cropping on the rhizospheric and endophytic bacterial community structure

**Fig. 4:**
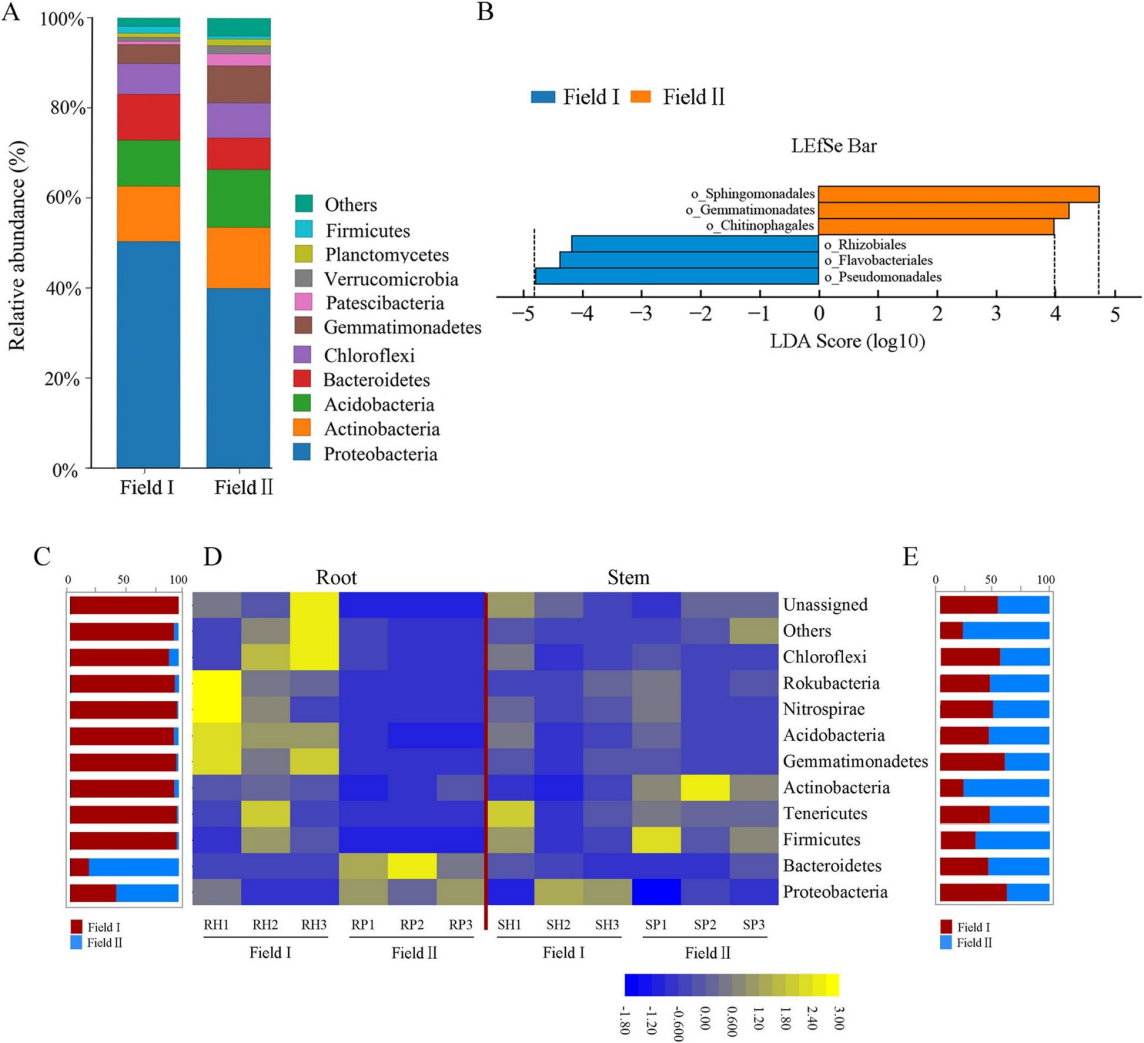
Bacterial communities in the rhizosphere and endophytic compartments of *A. mongholicus* plants grown in Field I and Field II. **A** Taxonomic classification of bacterial OTUs grouped at the phylum level. The two bars represent the relative abundance of bacterial phyla in the rhizosphere of *A. mongholicus* grown in Field I and Field II. Unclassified represents those species not taxonomically annotated. **B** The linear discriminant analysis effect size (LEfSe) algorithm screened out the specific bacteria at the order level present in the rhizosphere of *A. mongholicus* grown in Field I (blue) and Field II (orange). **C** The ratio in relative abundance of the top ten endophytic bacteria at the phylum level in *A. mongholicus* roots grown in Field I and Field II. **D** Heatmap analysis of the relative abundance of the top 10 endophytic bacteria at the phylum level in *A. mongholicus* roots and stems grown in Field I and Field II. **E** The percentage converted from the proportion of relative abundance of the top 10 endophytic bacteria at the phylum level in *A. mongholicus* stems grown in Field I and Field II

Bacterial phyla with relative abundance values > 10% were next analyzed. The dominant bacterial phyla in the rhizosphere were Proteobacteria, Actinobacteria, and Acidobacteria, which had relative abundances of 50.34%, 12.32%, and 10.22%, respectively, in Field I and 40.03%, 13.55%, and 12.85%, respectively, in Field II (Fig. 4A). The linear discriminant analysis [44] effect size (LEfSe) algorithm [44] was used to identify bacterial orders that were differentially abundant between the rhizosphere samples of *A. mongholicus* grown in the two fields. This analysis revealed three bacterial orders that explained most of the differences between the rhizosphere bacterial communities of plants grown in Field I and Field II (LDA log score threshold > 4.0 and *p* < 0.05). *Sphingomonadales* had the highest LDA score (4.72) in Field II, followed by *Gemmatimonadaceaes* (4.20), and *Chitinophagales* (4.00) (Fig. 4B). The indicator bacteria in Field I were the orders *Pseudomonadales* (4.76), *Flavobacteriales* (4.37), and *Rhizobiales* (4.15) (Fig. 4B).

The dominant bacterial phyla did not differ in the bulk soil compared to the rhizosphere soil (Fig. S3C). At the family level, LEfSe analysis showed that the bulk soil indicator bacteria in Field II were *Micrococcaceae* (4.56), *Pseudomonas* (4.27), *Rhizobiaceae* (4.13), and *Microbacteriaceae* (4.08), whereas in Field I they were the *Gemmatimonadaceae* (4.29) and *Nitrosomonadaceae* (4.12) (Fig. S3D). These results demonstrated the absence of *Rhizobiales* from the bulk virgin soil but not the continuously cropping soil; the reverse was true in the rhizosphere samples.

Analysis of the *A. mongholicus* root endophytic bacterial community revealed enrichment of Proteobacteria and Bacteroidetes but depletion of Firmicutes, Tenericutes, Actinobacteria, Gemmatimonadetes, Acidobacteria, Nitrospirae, and Rokubacteria among plants grown in Field II compared to Field I (Fig. 4C, D). In the stem samples, there was a slightly higher abundance of Actinobacteria among plants grown in Field II compared to Field I (Fig. 4D, E). All other phyla were similar in abundance between the stems of *A. mongholicus* grown in Field I and Field II (Fig. 4D, E). These results suggested that continuous cropping most strongly affected *A. mongholicus* root-associated bacterial communities.

## Effects of continuous cropping on *A. mongholicus* root exudates

**Fig. 5:**
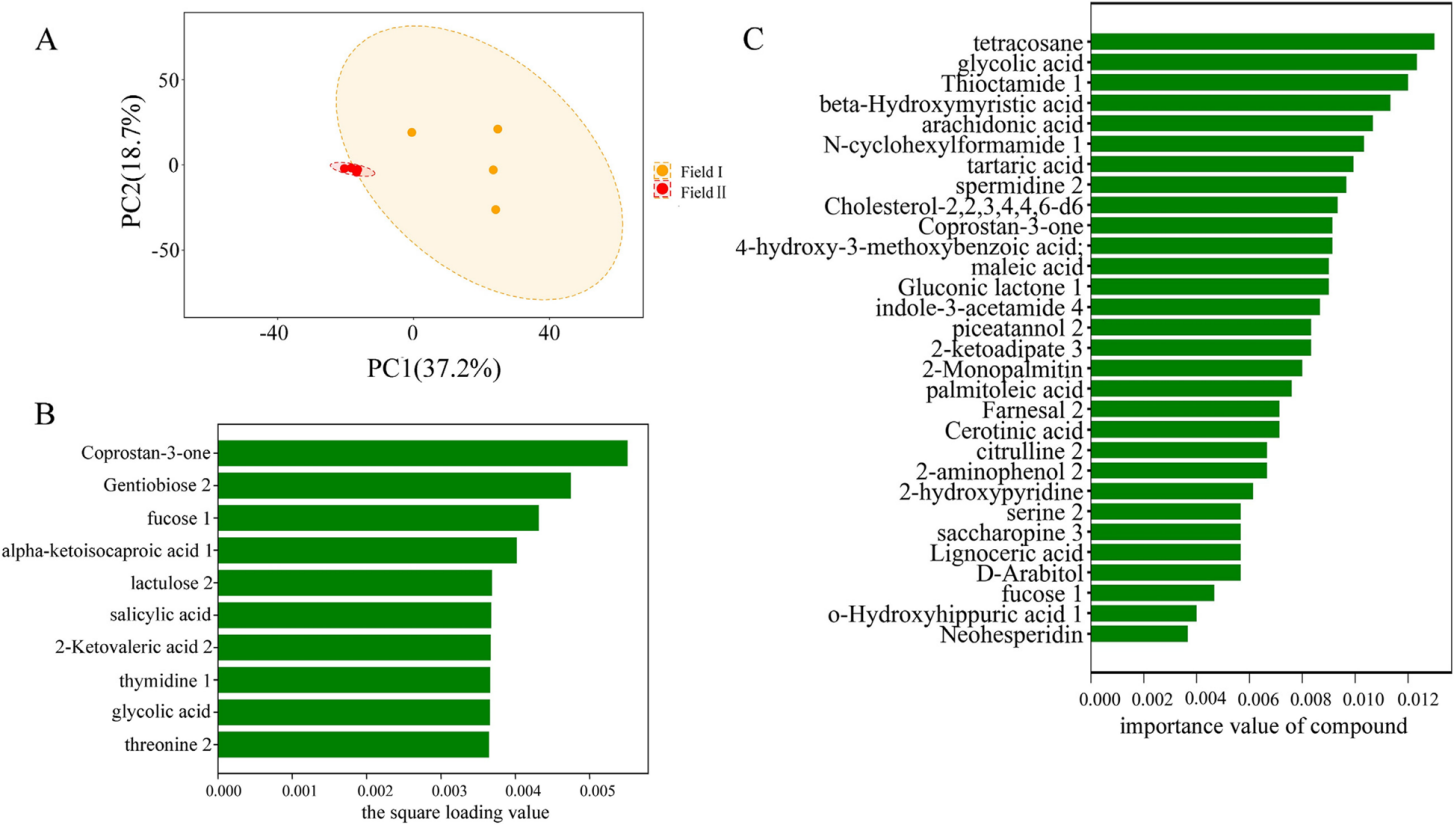
Analysis of metabolomics profiles in the rhizosphere soil of *A. mongholicus* planted under continuous cropping (Field II) and non-continuous cropping (Field I). **A** PCA plot of the metabolite profiles in rhizosphere soils from Field I and Field II obtained via GC–MS. The Adonis test had a *p*-value = 0.003). **B** The top 10 (top 4% of 236 total compounds) compounds sorted by their respective loading variable importance (in descending order) from the PCA of the two rhizosphere soil data sets. **C** The top 35 marker compounds were identified through a random forest classification of their relative abundances in the rhizosphere soil metabolomics profiles of *A. mongholicus* grown in Field I and Field II. The marker compounds are ranked in descending order of importance with respect to the accuracy of the model

To assess the potential functional effects of continuous cropping on plant–microbe interactions, we analyzed the *A. mongholicus* rhizosphere metabolome of plants grown in each field. Across all samples, a total of 550 chromatographic peaks were detected, corresponding to 240 identified compounds (Table S6). Identified metabolites included ketones, organic acids, phenols, amino acids, carbohydrates, amides, and alcohols. There were statistically significant differences in the relative abundances of 47 compounds between Field I and Filed II (p < 0.05) (Table S7). Specifically, 42 compounds were more abundant and five compounds were less abundant in the rhizosphere of *A. mongholicus* grown in Filed II compared to Field I. Levels of aniline-o-sulfonic acid and coprostan-3-one had the largest increase and decrease, respectively, in the rhizosphere of *A. mongholicus* grown in Field II compared to Field I. Principal component analysis (PCA) revealed clear differences between the rhizosphere metabolite profiles of plants grown in each field, with strong separation along PC1 (Fig. 5A), which explained 37.2% of the variance. To identify compounds that could best differentiate between the samples from each field, we extracted the top 10 compounds contributing to PC1 (Fig. 5B). In addition, we used a random forest classifier to select the top 30 compounds based on the loading variable importance scores (Fig. 5C).

**Fig. 6:**
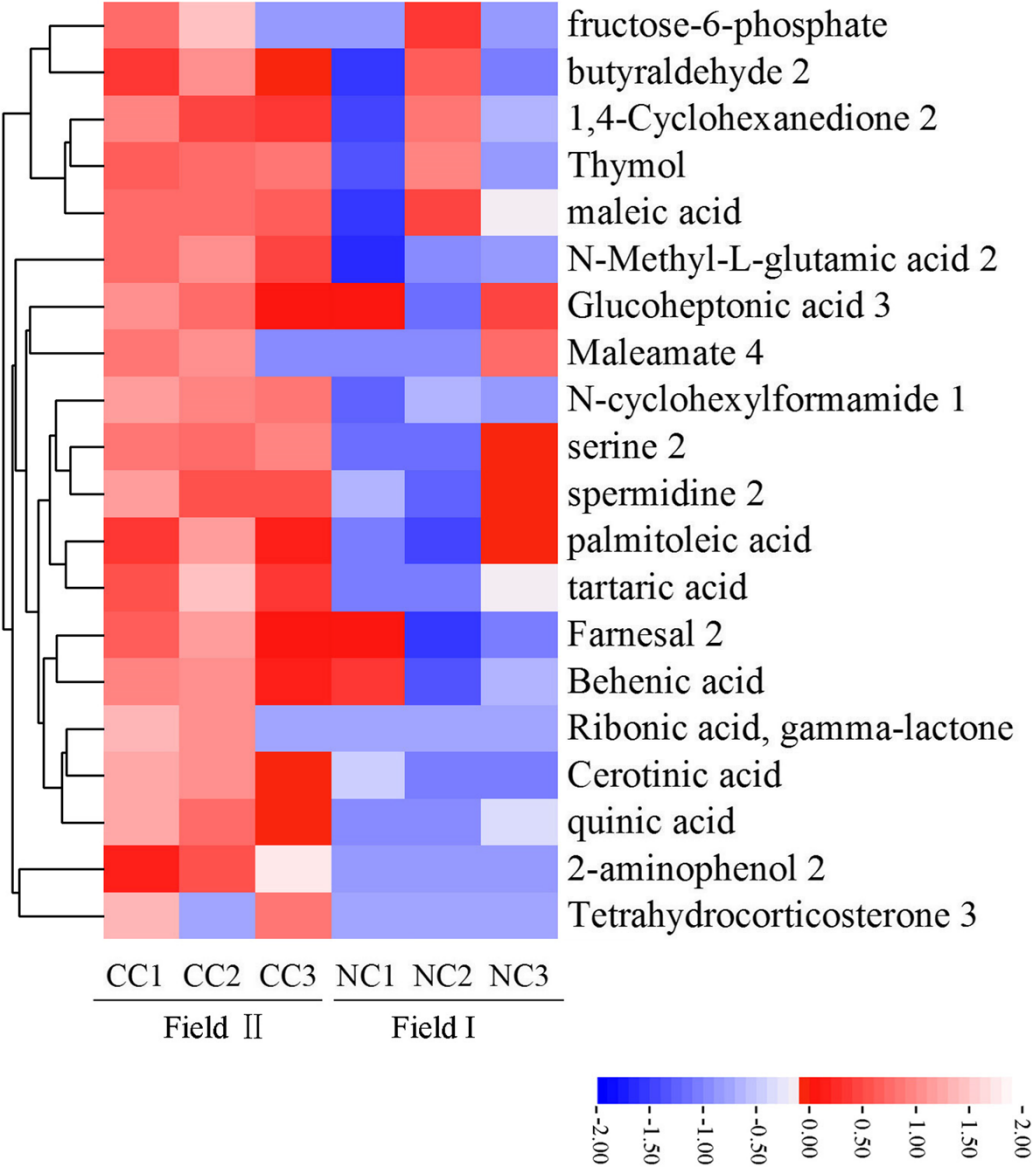
Composition and clustering of 20 compounds not only derived from the root exudates of *Astragalus* plants but whose relative content differed between the rhizosphere soils of *A. mongholicus* grown in Field I and Field II

Analysis of the rhizosphere soil alone could not identify plant-secreted compounds that were responsive to continuous cropping; metabolites extracted from the soil included not only plant root exudates but also microbial metabolites and compounds arising from the decomposition of plants, microbes, and soil organic matter [45, 46]. We therefore collected root exudates directly from *A. mongholicus* in a sterile hydroponic system. Gas chromatography (GC)—mass spectrometry (MS) analysis revealed the presence of 288 metabolites (Table S8) produced by the plants. To identify those that may have been involved in long-term continuous cropping challenges, we compared the 288 compounds extracted from *A. mongholicus* roots to the 47 metabolites that were differentially abundant between the Field I and Field II rhizosphere samples. There were 20 compounds that overlapped between the soil-derived and hydroponic datasets (Fig. 6; Table S9). Importantly, six of these metabolites (namely tartaric acid, maleic acid, palmitoleic acid, cerotinic acid, 2-aminophenol, and serine) were identified with PCA or random forest as some of the most differentially abundant metabolites between the rhizosphere samples collected from the two fields (Figs. 5B, C and 6). We therefore hypothesized that these 20 metabolites were likely responsive to continuous cropping.

To identify key metabolites that were most likely to be responsive to continuous cropping, we increased the stringency for screening differentially expressed metabolites to log2 (fold change) > 2 (Table S9). This yielded nine compounds: tetrahydrocorticosterone, 2-aminophenol, ribonic acid, quinic acid, fructose-6-phosphate, tartaric acid, cerotinic acid, maleamate, and serine (Fig. 6; Table S9). Tetrahydrocorticosterone and cerotinic acid are insoluble in water, which dissolve in a soluble organic solvent that may have certain effects on the growth and development of plants, and recent reports have indicated that plant roots exude organic acids under multiple stress conditions [47, 48], excluding ribonic acid, fructose-6-phosphate, and serine, we therefore selected 2-aminophenol, quinic acid, tartaric acid, and maleamate as candidate inhibitory metabolites for further analyses.

## Effects of candidate inhibitory metabolites on *A. mongholicus* root growth

**Fig. 7:**
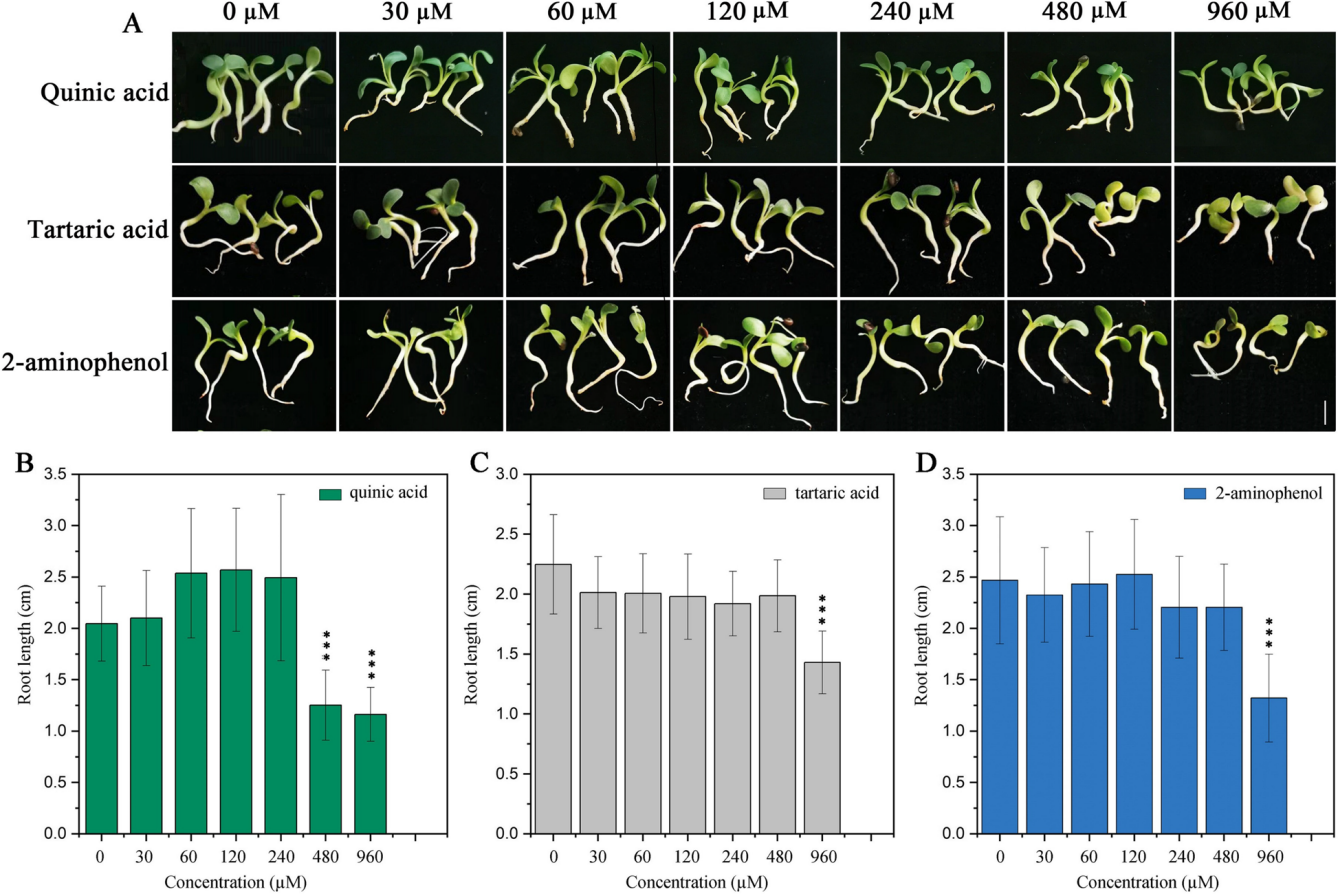
The morphology and root length of 10-day-old seedlings of *A. mongholicus* treated with different concentrations of 2-aminophenol, quinic acid, or tartaric acid. Seedling morphology (**A**), and root length (**B**, **C**, **D**). Values shown are the mean ± SD (*n* = 30), *** *p* < 0.001. Scale bar = 1 cm

We next sought to determine whether any of the candidate inhibitory metabolites were responsible for *A. mongholicus* root growth inhibition under continuous cropping conditions. To this end, we treated *A. mongholicus* seedling with varying concentrations of each compound individually. Indeed, exposure to 2-aminophenol, quinic acid, or tartaric acid inhibited root growth in a dosage-dependent manner (Fig. 7A). Specifically, exogenous application of 960 µmol L^−1^ 2-aminophenol or tartaric acid significantly suppressed root growth and caused chlorosis in the cotyledons (Fig. 7A, C, and D). Exogenous application of 480 µmol L^−1^ or 960 µmol L^−1^ quinic acids also significantly suppressed root growth (Fig. 7A, B). At a concentration of 960 µmol L^−1^, 2-aminophenol caused the most damage to plants and quinic acid caused the least, with tartaric acid falling in the middle. At all tested concentration, maleamate had negligible effects on *A. mongholicus* root growth (Fig. S5A, B). Thus, three of the four putative continuous cropping-responsive metabolites were demonstrated to inhibit *A. mongholicus* root growth.

## Effects of candidate inhibitory metabolites on *Fusarium oxysporum* mycelial growth

**Fig. 8:**
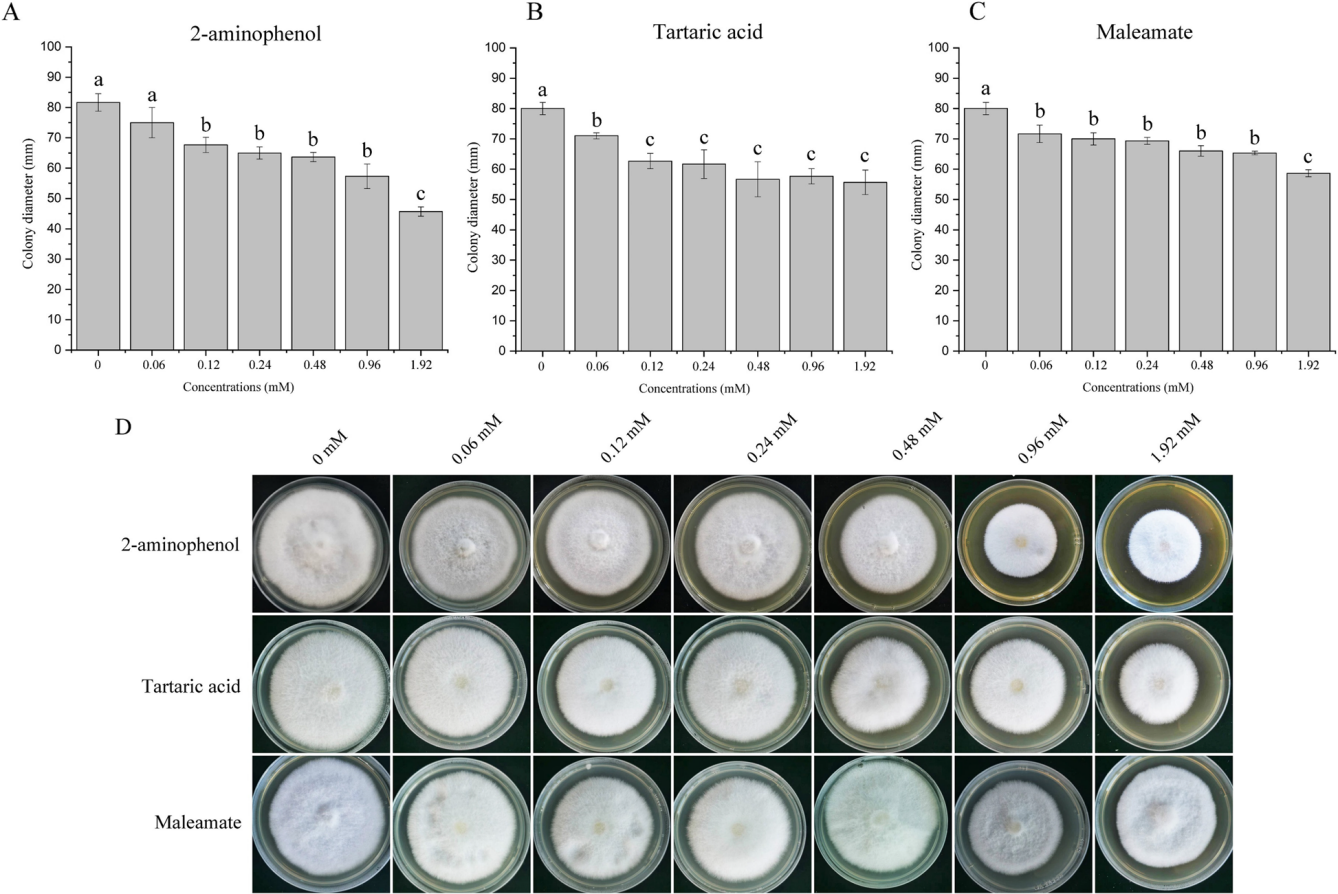
In vitro regulation of mycelial growth *Fusarium oxysporum* by 2-aminophenol, tartaric acid, and maleamate. Relative mycelial growth limited by 2-aminophenol, tartaric acid, or maleamate is shown as a percentage of fungal colonies on PDA media (**A**, **B**, **C**). Colony formation of *F. oxysporum* isolate HZ-F8 on PDA media supplemented with different doses (0, 0.06, 0.12, 0.24, 0.48, 0.96, and 1.92 mM) of 2-aminophenol, tartaric acid, and maleamate was investigated (**D**). Errors bars are the standard error for the means of five independent experimental replications. Mean values followed by the same letter are not significantly different at 5% level according to the LSD (least significant difference) test

Continuous *A. mongholicus* cropping is known to cause root rot disease, which is caused by *F*. *oxysporum* (Fig. S6) [49]. We therefore hypothesized that the candidate inhibitory metabolites may have affected *A. mongholicus* growth by altering *F*. *oxysporum* behavior. We therefore assessed the impacts of 2-aminophenol, quinic acid, tartaric acid, and maleamate on *F. oxysporum* growth. Radial mycelial growth was significantly suppressed by 2-aminophenol, tartaric acid, and maleamate (Fig. 8). Specifically, exogenous application of 1.92 mmol L^−1^ 2-aminophenol caused *F*. *oxysporum* colonies to grow to only half the size of untreated control colonies (Fig. 8A, D). Even lower concentration of tartaric acid and maleamate (0.06 mmol L^−1^) significantly inhibited *F. oxysporum* growth (Fig. 8B, C, D). However, quinic acid had no significant effects on mycelial growth (Fig. S7). Overall, these results were contrary to our expectation that metabolites with increased abundance under continuous cropping conditions would promote the observed increase in *Fusarium* associated with root rot disease.

## Discussion

Over the past several decades, continuous cropping obstacles have become a formidable problem due to their severe adverse impacts on crop yield and quality [50, 51]. Continuous cropping causes decreased yield among common crops, such as tomato (Solanum tuberosum) [52], ginseng (*P. ginseng*) [53], wheat (*Triticum aestivum*) [54], and fava bean (*Vicia faba*) [54, 55]. In the present study, continuous cropping of *A. mongholicus* for at least 20 years was shown to reduce biomass by 70–80% (Fig. 1), which constitutes crop failure. We conducted an integrated analysis of the microbial community structure and root exudate metabolites to discovered specific endophytic fungi and metabolites that may have driven disease in *A. mongholicus* seedlings under continuous cropping conditions.

Overall, root endophytic fungal diversity was considerably higher among *A. mongholicus* grown in continuously cropped soil compared to plants grown in virgin soil. These results were consistent with those another study showing that continuous cropping increase root endophytic fungal diversity [56]. However, the opposite was true of root endophytic bacteria (Fig. 2A, B). Thus, continuous *A. mongholicus* cropping augmented the diversity of both endophytic root fungi and bacteria, but in different ways.

*Rhizobiales* are well-studied bacterial symbionts of plants; in legumes, they commonly function as probiotics by fixing nitrogen and providing the resulting biologically available nitrogen to the host [33]. Three rhizobial strains (CCBAU 23380 T, CCBAU 23381, and CCBAU 23386) are known to be associated with *A. mongholicus* root nodules [57]. However, the *Rhizobiales diazotrophs* community composition has been shown to be influenced by cropping system types [58, 59]. Indeed, members of the order *Rhizobiales* were here found to be absent from the rhizosphere soil of continuously cropping *A. mongholicus*, even though they were present in the local bulk soil (Fig. 4B; Fig. S3D). *Rhizobiales* were thus specifically excluded from the rhizosphere community among continuously cultivated *A. mongholicus*. However, members of this order were found in the rhizosphere soil associated with *A. mongholicus* grown in virgin soil (Field I) (Fig. 4B; Fig. S3D). We therefore hypothesize that continuous cropping negatively affected *Rhizobiales* symbiosis with *A. mongholicus* roots, adversely affecting plant growth.

Ascomycota was the most abundant fungal phylum in the roots and stems of plants grown in both fields. However, the roots of plants grown in continuously cropped soil had a much higher relative abundance of *Fusarium* (Fig. 3C, D). This was notable because many *Fusarium* species are economically important plant pathogens [60]. A recent study similarly found that continuous cropping of *A. mongholicus* is associated with substantial accumulation of *Fusarium* species that cause root rot disease, such as *F. oxysporum* and *F. solani* [23]. Another study of root endophytic fungi in continuously cropped *P*. *notoginseng* showed high levels of potentially pathogenic members of the genera *Phoma*, *Collectotrichum*, and *Fusarium* [8]. In addition to *Fusarium*, we also found increased abundance of the genera *Cephalotrichum* [61], *Alternaria* [62], and *Paraphoma* [63], members of which may be pathogenic, in the roots of *A. mongholicus* plants grown in cultivated soil (Fig. 3C, D). Some species of fungal endophytes are harmful to their host and cause disease symptoms only when the host experiences stress conditions [64, 65]. We hypothesize that some fungal endophytes in this system operated as opportunistic pathogens of *A. mongholicus*, causing chlorosis, root necrosis, and biomass decline under continuous cropping, which functional as a stress condition.

The specific factors that led to enrichment of potentially pathogenic fungal species in Field II (the continuous cropping field) were not clear. Levels of available P and TC were both substantially higher in Field II than in Field I, whereas K was lower in Field II (Table S1). However, a recent study of peach rootstock found that the root fungal community is not significantly affected by soil P levels [66]. Increased soil TC and K slightly have been shown to slightly alter the endophytic fungal community structure in *Salix psammophila* and *Poa alsodes*, but not to report to promote accumulation of potentially pathogenic fungi [67, 68]. Thus, soil physicochemical factors were unlikely to be the cause of potential fungal pathogen enrichment within the roots of *A. mongholicus* grown in Field II.

Previous studies have found that autotoxic substances in root exudates are a major factor responsible for continuous cropping obstacles. We therefore conducted metabolomic analyses of the *A. mongholicus* rhizosphere and root exudates in sterile culture to shed additional light on crop failure under continuous cropping conditions. There were statistically significant differences in several key metabolites among rhizosphere samples collected from *A. mongholicus* grown in Field I compared to Field II (Fig. 5A). Because metabolites in the rhizosphere may be produced by either the plant or the associated microbial community, we focused on metabolites that were both differentially abundant between fields and confirmed via sterile plant culture to be produced by the plant itself. This yielded several metabolites that were produced by the plant and more abundant in the rhizosphere of plants grown in the continuous cropping field. These compounds (2-aminophenol, quinic acid, tartaric acid, and maleamate) were referred to as candidate inhibitory metabolites. Indeed, treatment with any one of the former three compounds inhibited *A. mongholicus* root growth in a dose-dependent manner (Fig. 7A). This was consistent with prior studies showing that phenolic acids are the primary substances causing autotoxicity in Lettuce root exudates; these compounds have been shown to limit plant growth, reducing yield [69]. For example, strawberry root exudates contain benzoic acid, a potent growth inhibitor [70]. Our results therefore suggest that *A. mongholicus* growth in Field II was inhibited due to an accumulation of toxic root exudates, likely including 2-aminophenol, quinic acid, and tartaric acid.

Root exudates secreted into the rhizosphere regulate not only plant performance, but also the soil microbial community structure. Due to the increased abundance of 2-aminophenol, quinic acid, maleamate, and tartaric acid under continuous cropping conditions, we wondered whether these plant-produced metabolites may have affected *Fusarium* growth. Tests of the four compounds on cultured *F. oxysporum* revealed varying degrees of hyphal growth suppression in response to treatment with 2-aminophenol, tartaric acid, or maleamate. Thus, 2-aminophenol and tartaric acid inhibited both plant and fungal growth; the other two candidate inhibitory metabolites performed only one of these functions each, inhibiting growth of only the plant (quinic acid) or only *F. oxysporum* (maleamate). This is the first report detailing accumulation of toxic root exudates in the rhizosphere soil of *A. mongholicus* grown under long-term continuous cropping conditions, and the first report of such compounds also inhibiting *F. oxysporum* hyphal growth.

Based on these results, we posit that years of continuous *A. mongholicus* cropping led to enrichment of phytopathogens in the soil, causing *A. mongholicus* to secrete defensive compounds such as 2-aminophenol and tartaric acid. However, long-term accumulation of these compounds led to a counterproductive impairment of plant growth, which could explain the decreased plant performance under continuous cropping conditions. Root exudates can also have negative effects on plant growth by promoting soil-borne disease [28], which would compound the growth inhibition; although we were unable to identify compounds in *A. mongholicus* root exudates that stimulated *F*. *oxysporum* growth. Further studies addressing the mechanisms by which root exudates interact with the endophytic and rhizosphere microbiomes would provide a strong scientific basis for development of techniques to control continuous cropping obstacles, allowing optimization of agricultural productivity and ensuring crop health.

## Conclusion

Analyses of *A. mongholicus* growth, root endophytic and rhizosphere microbiota, and root-associated metabolites were conducted to identify differences between plants grown in virgin soil and those grown in a long-term continuous cultivation field. Plants grown under continuous cropping conditions showed significantly decreased fresh and dry weight. Importantly, potentially pathogenic fungi were enriched in the roots of *A. mongholicus* grown under continuous cropping conditions. A total of 20 metabolites in the *A. mongholicus* root exudates were differentially abundant between rhizosphere samples collected from the two fields. Four of those compounds (2-aminophenol, quinic acid, tartaric acid, and maleamate) were found to inhibit the growth of *A. mongholicus*, the soil-borne pathogen *F. oxysporum*, or both. These results provide compelling evidence that continuous cropping impaired *A. mongholicus* growth and significantly altered the root-associated microbiome and root exudate composition. This comprehensive analysis enhances our understanding of the ways in which continuous cropping affects *A. mongholicus*, offering valuable new information that can be used in future studies to design effective, economical approaches to achieve food security.

## Materials and methods

### Study sites and *A. mongholicus* planting

The study sites comprised one field with no history of *A. mongholicus* cultivation (Field I) and one field with ≥ 20 years of continuous *A. mongholicus* cropping (Field II) in Lujiamen Village, Longxi County, Gansu Province, China (35º04ʹ N, 104º36ʹ E). The two fields were under the same conditions (chemical fertilizer applications, temperature, and light intensity). The soil in this region was classified as loam, with an annual mean precipitation of 445.8 mm and a temperature of 7.7 ℃. Additionally, there were approximately 2,292 h of sunshine per year. The physical characteristics of the two fields were as closely as possible (Table S1).

One-year-old *A. mongholicus* individuals of uniform size were plated 20 cm apart in both fields that had an area of 800 square meters in March 2020 and harvested in August 2020. The two fields were treated with identical management practices. According to the above planting pattern, in 2019, we also have observed *A. mongholicus* growth cultivated in the fields adjacent to Field I with no history of *A. mongholicus* to compare with Field II.

## Morphological phenotypes of *A. mongholicus* plants

The whole *A. mongholicus* plants were harvested from two fields and removed from the junction of their roots and stems using scissors. The differences in root health between the two fields, and then a 10-cm segment of the stem (beginning from the root-stem junction) was obtained to characterize either its cross-sectional or longitudinal structure.

### Plant and soil collection and processing

#### Biomass measurements

There were two fields, each with 20 replicates were collected on 22 August, 2020. To ensure the random acquisition of plants, we selected one plant per every 2 square meters. For all selected plants, a shovel was used to remove the entire plant, including the root system, from the ground. Each plant was separated into the aerial tissue (the leaves, stems and branches) and the below-ground tissue (the roots). The two tissue types were transported to the lab and immediately measured to record the fresh weight, then oven dried at 85 °C for 72 h before measuring the dry weight.

#### Rhizosphere soil collection

In the remaining analyses, the plants were harvested from the soil using a shovel. For each selected plant, the soil adhered to the roots was gently removed and retained as the rhizosphere soil. The plants were processed as outlined below (*plant tissue processing*). A total of 14 rhizosphere soil samples were acquired from two different fields, each encompassing seven samples. transported on ice to the laboratory, and aliquots of approximately100 mg were weighed out. For each field, three out of the seven rhizosphere soil samples were randomly replicated for microbial community analysis, adhering to the description provided below. These soil samples were passed through a 2-mm sieve and stored at -80℃. The other four samples were stored at 4℃ before the metabolomic analysis outlined below.

#### Bulk soil collection

At a 30-cm distance from the roots of chosen plants in Field I and Field II, a total of 14 bulk soil samples were obtained using a shovel at depth of 0—10 cm, each encompassing seven bulk soil samples, A total of 14 bulk soil samples were transported on ice to the laboratory and separated into aliquots of approximately100 mg. For each field, three out of the seven bulk soil samples were randomly utilized for biological replication, passed through a 2-mm sieve, and stored at -80℃ prior to microbial community analysis. The other four samples were stored at 4℃ before assessing soil physicochemical property measurements as described below.

#### Plant tissue processing

Nine plant samples were harvested from each field, with seven from the rhizosphere soil collection segment and two plants dug out of the field where seven plants were previously harvested. Nine plants were separated into three replicates, with each replicate composed of three plants. To collect the root and stem tissues, approximately170 mg of tissue was obtained from the top, middle, and bottom segments of the roots (ratio 1:1:1). Stems were separated into 1-cm segments beginning at 2 cm from the junction of the roots and stem. This resulted in approximately 500 mg of root and stem tissue per replicate. All tissues were washed using tap water, sterilized with 75% ethanol for 30 s, followed by 3% sodium hypochlorite for 5 min, and washed three times with sterile distilled water. The root and stem samples were then cut into small segments (5 × 5 × 5 mm) and stored in 5-mL vials at -80℃ until prior to further processing. Subsamples were not frozen, placed on nutrient agar plates, and incubated at 37 °C for two days to guarantee effective sterilization.

### Soil physicochemical property measurements

Each soil sample was mixed with water at a ratio of (w/v) and shaken at 50 rpm for 30 min to mix. The pH was measured with a PHS-3C pH meter (Shanghai INESA Instrument Co., Ltd., shanghai, China). Eight subsamples were air dried and passed through 2-mm mesh prior to analysis of soil properties as previously described [71]. Briefly, the soil total carbon (TC) and total nitrogen [72] were quantified using a CN Analyzer (Vario Max CN, Elementar, Hanau, Germany). Available phosphorus [73] and K were assessed with a UV–Vis spectrophotometer (UV-2450, Shimadzu, Japan).

### Microbial community analyses

#### DNA extraction

The total DNA was extracted from each dedicated rhizosphere and bulk soil replicates (0.5 g each) using the PowerSoil DNA Isolation kit (Mo_Bio Laboratories, Carlsbad, CA, USA) following the manufacturer’s instructions. DNA was extracted from 0.5 g samples of surface sterilized roots and stems with the NucleoSpin 96 Soil Kit (Takara Bio USA, San Jose, CA, USA) following the manufacturer’s instructions. DNA quality was assessed with a 1.8% agarose gel and quantity was measured with a Nanodrop2000 spectrophotometer (Thermo Fisher Scientific, Wilmington, DE, USA).

#### PCR and sequencing library preparation

The internal transcribed spacer region 1 (ITS1) of the fungal rRNA gene was amplified from three samples each of rhizosphere soil, bulk soil, roots, and stems collected from each of the two fields. PCR was conducted with the forward primer ITS1F (5'-CTTGGTCATTTAGAGGAAGTAA-3') and the reverse primer ITS2 (5'-GCTGCGTTCTTCATCGATGC-3'), which produce strips between 200 and 400 bp amplicon. Each primer contained an 8-bp error-correcting barcode unique to each sample. There were three technical replicates per sample. Thermal cycling conditions were as follows: 94 °C for 5 min; 35 cycles of 95 °C for 30 s, 56 °C for 1 min, and 72 °C for 40 s; 72 °C for 7 min. The resulting technical replicates for sample were pooled and purified using the E.Z.N.A.® Gel Extraction Kit (Omega Bio-Tek, Norcross, GA, USA). The purified PCR products were run on a 1.8% agarose gel. Clearly defined bands between 350 and 550 bp were extracted using Monarch® DNA Gel Extraction kit (NEB #T1020) and combined for sequencing on the Illumina HiSeq 2500 platform.

The V3-V4 region of the 16S rRNA gene was amplified from the same set of DNA samples. For samples extracted from soil, the primers 338F (5ʹ-ACTCCTACGGGAGGCAGCA-3ʹ) and 806R (5ʹ-GGACTACHVGGGTWTCTAAT-3') were used; for stem and root samples, the primers 335F (5'-CADACTCCTACGGGAGGC-3') and 769R (5'-ATCCTGTTTGMTMCCCVCRC-3') were used. Each 50 µL reaction mixtures contained 40 ng of template. The purified PCR products were run on a 1.8% agarose gel. Clear bands between 290 and 310 bp were combined for sequencing. The PCR products were then mixed and sequenced on an Illumina HiSeq 2500 platform with the same sequencing information as previously reported.

#### Raw sequencing data processing and analysis

The raw paired-end reads were merged using FLASH v1.2.1. Low-quality merged reads were removed using Trimmomatic v0.33 and chimeric sequences were identified and discarded with UCHIME v8.1. The resulting high-quality reads were clustered into OTUs at the standard 97% similarity threshold using USEARCH v10.0. OTUs with an abundance ≤ 0.005% were removed. Taxonomy was assigned to the representative sequences for each OUT using the Unite database (release 7.2, http://unite.ut.ee/index.php) [74] for fungi and Silva (Release128, http://www.arb-silva.de) [75] for bacteria.

#### Statistical analysis

Statistically significant differences in biomass, alpha diversity, phylum, genus, root length of *A. mongholicus* and the fungal radial growth were examined with ANOVA test in SPSS software (version 20.0 SPSS). Alpha diversity (Shannon index) was calculated using Mothur version.1.30 (http://www.mothur.org/). The relative abundance of certain fungal and bacterial taxa was characterized by analyzing the classification results, via the ratio of the number of sequences belonging to given fungal and bacterial taxa in the sample relative to the total number of sequences in the sample using QIIME (https://qiime2.org). Principal component analysis (PCA) was conducted using R (www.r-project. org). Linear discriminant analysis integrated with an effect size measurements (LEfSe) analysis was performed to identify significantly different (P < 0.05) taxa between the two groups, with an LDA score of at least 4.0 [76].

#### Metagenomic sequencing

This was accomplished with high-throughput sequencing of the internal transcribed spacer (ITS) region to identify fungal community and the 16S rRNA gene to identify bacterial community. Sequencing yielded a total of 5,947,274 high-quality, nonchimeric sequences, comprising 4,756,850 fungal and 1,190,424 bacteria reads. The median numbers of fungal and bacterial sequences per sample were 198,202 (range = 36,166–444,272) and 49,601 (range = 30,301–74,003), respectively (Table S2 and S3). The rarefaction curves of all samples approached saturation, indicating that the sequencing depth captured the fungal and bacterial diversity sufficiently to conduct a robust analysis (Fig. S2A, B). Sequencing data for each sample were rarefied to the lowest per-sample read count (36,166 for fungi and 30,301 for bacteria). At a threshold of 97% similarity, the sequences were clustered into a total of 961 fungal and 8,326 bacteria operational taxonomic units (OTUs) (Table S4 and S5), corresponding to Good’s coverage values of 99.99% ± 0.002% and 99.70% ± 0.026%, respectively.

### Metabolomic analyses

#### Soil extractions

Rhizosphere soils samples were collected and processed as described above (Plant and soil collection and processing). Aliquots (1 g each) were transferred to 5-mL tubes containing 1 mL of cold extraction mixture (3:1 methanol: dH2O [v/v]), 1 mL of ethyl acetate, and 5 µL of an internal standard (0.5 mg/mL adonitol). Samples were vortexed for 30 s, homogenized with a ball mill for 4 min at 35 Hz, and ultrasonicated for 5 min in ice water. The vortex, homogenization, and ultrasonication steps were then repeated twice. After centrifugation at 4 °C and 10,000 rpm for 15 min, the supernatant was transferred to a 5-mL Eppendorf tube for a two-phase extraction. Each sample was mixed with 1 mL of cold extraction mixture and 1 mL of ethyl acetate. For each sample, 2.5 mL of supernatant was carefully transferred to a fresh tube; 500 µL of each sample was combined into a single quality control (QC) sample. Each sample was then evaporated in a vacuum concentrator. After evaporation, 30 µL of methoxyamination hydrochloride (20 mg/mL in pyridine) was extract the rhizosphere soil metabolite at 80℃ for 30 min. Samples were derivatized at 70 °C for 1.5 h with the addition of 40 µL N, O-bis (trimethylsilyl) trifluoroacetamide regent (1% trimethylsilyl chloride, [v/v]). All samples were then cooled to room temperature. Fatty acid methyl ester in chloroform (5 µL) was added to the QC sample; then all samples were analyzed with GC-time-of-flight (TOF)-MS as described below (*GC- TOF- MS*).

#### Root exudate extraction

*A. mongholicus* seeds were surface-sterilized with 3% sodium hypochlorite solution for 5 min, washed with 75% alcohol for 1 min, then washed three times in deionized water. Sterilized seeds were placed in a petri dish (90 mm in diameter) lined with sterilized filter paper wetted with 5 mL of sterilized distilled water. The seeds were incubated for 10 d at 22 °C under a 16/8 h light/dark cycle to germinate. Uniformly germinated seedlings were then moved into each of four pots containing soil collected from Field I (virgin soil), with six plants per pot. Seedlings were grown for 10 months; then 12 plants were selected at random for collection. The roots were washed well with deionized water, transferred to sterile Hoagland solution [77], and gently shaken at 80 rpm for 2 h each day. The plants were then placed in 1-L cylinders (each containing four plants) filled with 1 L of sterilized distilled water and completely wrapped the cylinder in aluminum foil. Plants were incubated for 3 d at 22 °C under a 16/8 h light/dark cycle with gentle shaking as described above. The resulting root exudates in sterile water were passed through sterile filters (0.2 µm pore size) to remove root debris and stored at -20 °C, and then dried with a lyophilizer (LGJ-10 Beijing Songyuanhuaxing Technology Develop Co., Ltd., China). After evaporation, the freeze-dried root exudates were transferred to 2 mL EP tube and extracted with 1 mL of extraction solution (*V*_methanol_: *V*_water_ = 3:1). Additionally, 5 µL of adonitol (0.5 mg/mL stock in water) was added as the internal standard. The contents were then mixed for 30 s by vortexing. Subsequently, the mixtures were homogenized in a ball mill at 45 Hz for 4 min, ultrasonicated for 5 min (while incubating in ice water), centrifuged at 13,000 rpm and at a temperature of 4℃ for 15 min. The supernatant (0.75 mL) was subsequently transferred to a new 2 mL GC/MS glass vial. After completely drying in a vacuum concentrator without heating, 30 µL of Methoxyamination hydrochloride (20 mg/mL in pyridine) was extract the root exudation at 80℃ for 30 min, then derivatized at 70 °C for 1.5 h by the addition of 40 µL N,O-bis(trimethylsilyl) trifluoroacetamide regent (1% trimethylsilyl chloride, v/v). All samples were then cooled to room temperature. All samples were analyzed using the GC-TOF–MS process described below.

#### GC-TOF–MS

Rhizosphere soil metabolites and root exudate metabolites were analyzed via GC-TOF–MS. This was conducted on an Agilent 7890 gas chromatograph coupled with a TOF–MS device equipped with a DB-5MS capillary column (30 m × 250 µm × 0.25 µm). Each 1 µL injection was performed using a 10/1 split ratio. The front injection temperature was 280 °C and the column flow rate was 1 mL/min. The oven temperature started at 50 °C, increased by 10 °C /min until it reached to 310 °C, and then was held for 8 min. Total ion scans were collected from 50–500 amu. The data were processed using Chroma TOF v4.3x(LECO Corporation, St. Joseph, MI, USA) [78] and the LECO-Fiehn Rtx5 database [79].

### *A. mongholicus* treatment with candidate inhibitory metabolites

The candidate inhibitory compounds 2-aminophenol (CAS 95–55-6), quinic acid (CAS 77–95-2), tartaric acid (CAS 526–83-0), and maleamate (CAS 557–24-4) were purchased from Lanzhou Tianhong Yihua Biotechnology Co., LTD and diluted in sterilized water to working concentrations of 30, 60, 120, 240, 480, and 960 µmol L^−1^. *A. mongholicus* seeds were surface-sterilized with 75% ethanol for 30 s and 3% sodium hypochlorite for 5 min, and then washed five times with sterilized water. A single sterilized seed was placed in each of 20 petri dishes (12 cm × 12 cm) containing two layers of Whatman filter paper moistened with sterilized water. After three days, 40 uniformly germinated seeds were selected and placed in each petri dish (12 cm × 12 cm) containing two layers of Whatman filter paper moistened with 10 mL of sterilized water or the working concentration of a candidate inhibitory metabolite, producing a total of 84 petri dishes. There were three biological replicates for each concentration of each compound. Root lengths were measured 10 days after germination. For each dish, 10 mL of the appropriate treatment solution was added to the filter paper every 3 days. This experiment was repeated three times.

### *F. oxysporum* treatment with candidate inhibitory metabolites

*F. oxysporum* was isolated from the diseased roots of *A. mongholicus* plants grown in Field II and designated HZ-F8. Plants grown in pots and inoculated with *F.* oxysporum HZ-F8 showed a high disease incidence, with affected plants displaying typical root rot symptoms (Fig. S6); HZ-F8 was thus appropriate for further experiments. The fungus was cultured on potato dextrose agar (PDA) medium at 28 °C for 7 days in the dark. To establish whether the candidate inhibitory metabolites affected fungal mycelial growth, mycelial discs of 8 mm in diameter were cut from the growing edge of 7-day-old fungal colonies with uniform growth. PDA plates (1/2-strength) were prepared that contained 2-aminophenol, quinic acid, tartaric acid, or maleamate at the concentrations described above for *A. mongholicus* treatment with candidate inhibitory metabolites. The mycelial discs were placed upside-down in the center of these plates and cultured at 28 °C for 7 day in the dark. Colony diameters were then measured and the relative fungal colony size was expressed as a percentage (%) of the untreated control size.

### Supplementary Information

**Supplementary Material 1.**

**Supplementary Material 2.**

**Supplementary Material 3.**

**Supplementary Material 4.**

**Supplementary Material 5.**

## Data Availability

All data supporting the findings of this study are available in the Supplementary Information. The 18S rRNA gene sequences were deposited into the NCBI Sequence Read Archive (accession number: PRJNA875632); the 16S rRNA gene sequences were provided and are available at the NCBI Sequence Read Archive (SRP) repository (accession number: PRJNA875504).
