# Supplementary material for: Impacts of continuous cropping on the rhizospheric and endospheric microbial communities and root exudates of Astragalus mongholicus
Source: BMC Plant Biol. 2024 Apr 26;24:340. doi: 10.1186/s12870-024-05024-5 (PMC11047024; doi:10.1186/s12870-024-05024-5)
Supplement: Supplementary file 1 — Supplementary Material 1. [file 12870_2024_5024_MOESM1_ESM.docx]

| **Table S1 The physico-chemical properties (mean±SE, n=4) of bulk soil of *A. mongholicus*** | | | | | |
| --- | --- | --- | --- | --- | --- |
|  | pH | Avail-P (mg/kg) | Avail-K (mg/kg) | TC (%) | TN (%) |
| FieldⅠ | 8.20 ± 0.22 | 21.61 ± 0.69 | 163.33 ± 4.16 | 2.28 ± 0.02 | 0.91 ± 0.01 |
| FieldⅡ | 8.13 ± 0.20 | 63.23 ± 1.89 | 113.66 ± 4.16 | 2.50 ± 0.02 | 1.19 ± 0.12 |
| Sig. | NS | ** | ** | ** | NS |
| TC total carbon content, TN total nitrogen content, Sig. significance  ** P < 0.01; NS, P > 0.05  ·^-1^  **Soil physicochemical properties**  We next assessed the physicochemical properties of the soil in each field to identify factors that may have been responsible for the differences in *A. mongholicus* health. The soil pH was similar between Field I and Field II (Table S1). There was significantly more available P in Field II than in Field I (by 2.92-fold), but less available K (K) (Table S1). The total C (TC) content of Field II significantly exceeded that of Field I (Table S1). Total N levels were not significantly different between the fields (Table S1). Thus, differences in total N or pH could not have been responsible for the decreased health of plants grown in Field II compared to Field I, whereas differences in available P, K, or TC may have played a role. | | | | | |

**Supporting Tables and Figures**

| **Table S2 The sequence number of effective reads of fungi generated in all samples** | | | |
| --- | --- | --- | --- |
| Compartment | Field type | Replicates | Seq_num |
| Bulk soil | FieldⅠ | 1 | 51270 |
| Bulk soil | FieldⅠ | 2 | 83493 |
| Bulk soil | FieldⅠ | 3 | 78042 |
| Bulk soil | FieldⅡ | 1 | 67813 |
| Bulk soil | FieldⅡ | 2 | 70172 |
| Bulk soil | FieldⅡ | 3 | 70598 |
| Rhizosphere | FieldⅠ | 1 | 65058 |
| Rhizosphere | FieldⅠ | 2 | 66357 |
| Rhizosphere | FieldⅠ | 3 | 36166 |
| Rhizosphere | FieldⅡ | 1 | 74991 |
| Rhizosphere | FieldⅡ | 2 | 72040 |
| Rhizosphere | FieldⅡ | 3 | 71026 |
| Root | FieldⅠ | 1 | 443006 |
| Root | FieldⅠ | 2 | 444272 |
| Root | FieldⅠ | 3 | 362396 |
| Root | FieldⅡ | 1 | 329039 |
| Root | FieldⅡ | 2 | 250051 |
| Root | FieldⅡ | 3 | 401012 |
| Stem | FieldⅠ | 1 | 302056 |
| Stem | FieldⅠ | 2 | 229917 |
| Stem | FieldⅠ | 3 | 322307 |
| Stem | FieldⅡ | 1 | 250202 |
| Stem | FieldⅡ | 2 | 348568 |
| Stem | FieldⅡ | 3 | 266998 |

| **Table S3 The sequence number of effective reads of bacteria generated in all samples** | | | |
| --- | --- | --- | --- |
| Compartment | Field type | Replicates | Seq_num |
| Bulk soil | FieldⅠ | 1 | 53831 |
| Bulk soil | FieldⅠ | 2 | 74003 |
| Bulk soil | FieldⅠ | 3 | 62655 |
| Bulk soil | FieldⅡ | 1 | 52327 |
| Bulk soil | FieldⅡ | 2 | 63305 |
| Bulk soil | FieldⅡ | 3 | 69066 |
| Rhizosphere | FieldⅠ | 1 | 51914 |
| Rhizosphere | FieldⅠ | 2 | 37816 |
| Rhizosphere | FieldⅠ | 3 | 55756 |
| Rhizosphere | FieldⅡ | 1 | 31935 |
| Rhizosphere | FieldⅡ | 2 | 30301 |
| Rhizosphere | FieldⅡ | 3 | 31777 |
| Root | FieldⅠ | 1 | 48064 |
| Root | FieldⅠ | 2 | 35205 |
| Root | FieldⅠ | 3 | 52044 |
| Root | FieldⅡ | 1 | 50153 |
| Root | FieldⅡ | 2 | 58788 |
| Root | FieldⅡ | 3 | 52437 |
| Stem | FieldⅠ | 1 | 33563 |
| Stem | FieldⅠ | 2 | 59575 |
| Stem | FieldⅠ | 3 | 51463 |
| Stem | FieldⅡ | 1 | 32420 |
| Stem | FieldⅡ | 2 | 53029 |
| Stem | FieldⅡ | 3 | 48997 |

| **Table S4 Statistics on features of the high-throughput sequencing-based analysis of Fungal communities of each sample** | | | | | | | | |
| --- | --- | --- | --- | --- | --- | --- | --- | --- |
| Compartment | Number  of  sequence | Number  of  OTU | Number  of  genera | Number  of  family | Number  of  order | Number  of  class | Number  of  phylum | Coverage |
| Bulk soil  (FieldⅠ) | 70935 | 164 | 69 | 43 | 24 | 12 | 4 | 0.999 |
| Bulk soil  (FieldⅡ) | 69528 | 172 | 78 | 43 | 27 | 13 | 5 | 0.999 |
| Rhizosphere  (FieldⅠ) | 55860 | 181 | 82 | 46 | 30 | 13 | 5 | 0.999 |
| Rhizosphere  (FieldⅡ) | 72686 | 135 | 66 | 36 | 24 | 12 | 4 | 0.999 |
| Root  (FieldⅠ) | 416558 | 71 | 36 | 25 | 19 | 10 | 3 | 1.000 |
| Root  (FieldⅡ) | 326700 | 75 | 39 | 27 | 20 | 10 | 4 | 0.999 |
| Stem  (FieldⅠ) | 284760 | 94 | 47 | 31 | 22 | 11 | 4 | 1.000 |
| Stem  (FieldⅡ) | 288589 | 69 | 32 | 25 | 19 | 10 | 3 | 0.999 |

| **Table S5 Statistics on features of the high-throughput sequencing-based analysis of Bacterial communities of each sample** | | | | | | | | |
| --- | --- | --- | --- | --- | --- | --- | --- | --- |
| Compartment | Number  of  sequence | Number  of  OTU | Number  of  genera | Number  of  family | Number  of  order | Number  of  class | Number  of  phylum | Coverage |
| Bulk soil  (FieldⅠ) | 63496 | 1580 | 432 | 246 | 162 | 81 | 28 | 0.998 |
| Bulk soil  (FieldⅡ) | 61566 | 1532 | 418 | 237 | 155 | 79 | 25 | 0.998 |
| Rhizosphere  (FieldⅠ) | 48495 | 1446 | 418 | 239 | 159 | 79 | 28 | 0.997 |
| Rhizosphere  (FieldⅡ) | 31338 | 1404 | 387 | 225 | 149 | 77 | 26 | 0.995 |
| Root  (FieldⅠ) | 45104 | 739 | 349 | 160 | 88 | 34 | 14 | 0.996 |
| Root  (FieldⅡ) | 53793 | 385 | 219 | 113 | 69 | 27 | 11 | 0.997 |
| Stem  (FieldⅠ) | 48200 | 620 | 306 | 145 | 82 | 33 | 14 | 0.997 |
| Stem  (FieldⅡ) | 44815 | 620 | 311 | 147 | 81 | 31 | 14 | 0.996 |


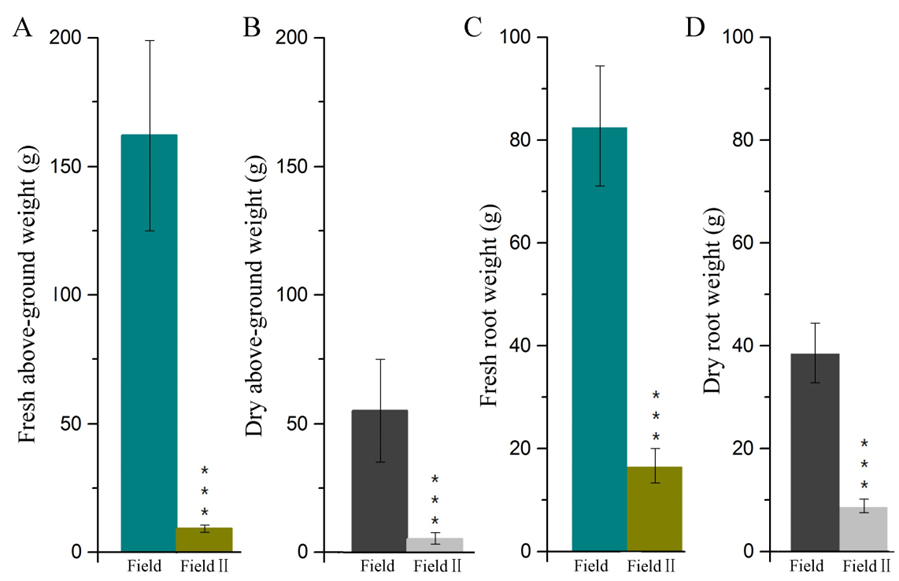


**Fig. S1.** Biomass of *A. mongholicus* plants grown in virgin soil (Field) or under continuous cropping conditions (FieldⅡ). Quantification of the fresh and dry weight of above-ground tissue (A, B) and roots (C, D) collected from *A. mongholicus* plants grown in Field and FieldⅡin 2019. (n = 20 plants per field). ***p < 0.001 (Student’ s t-test).


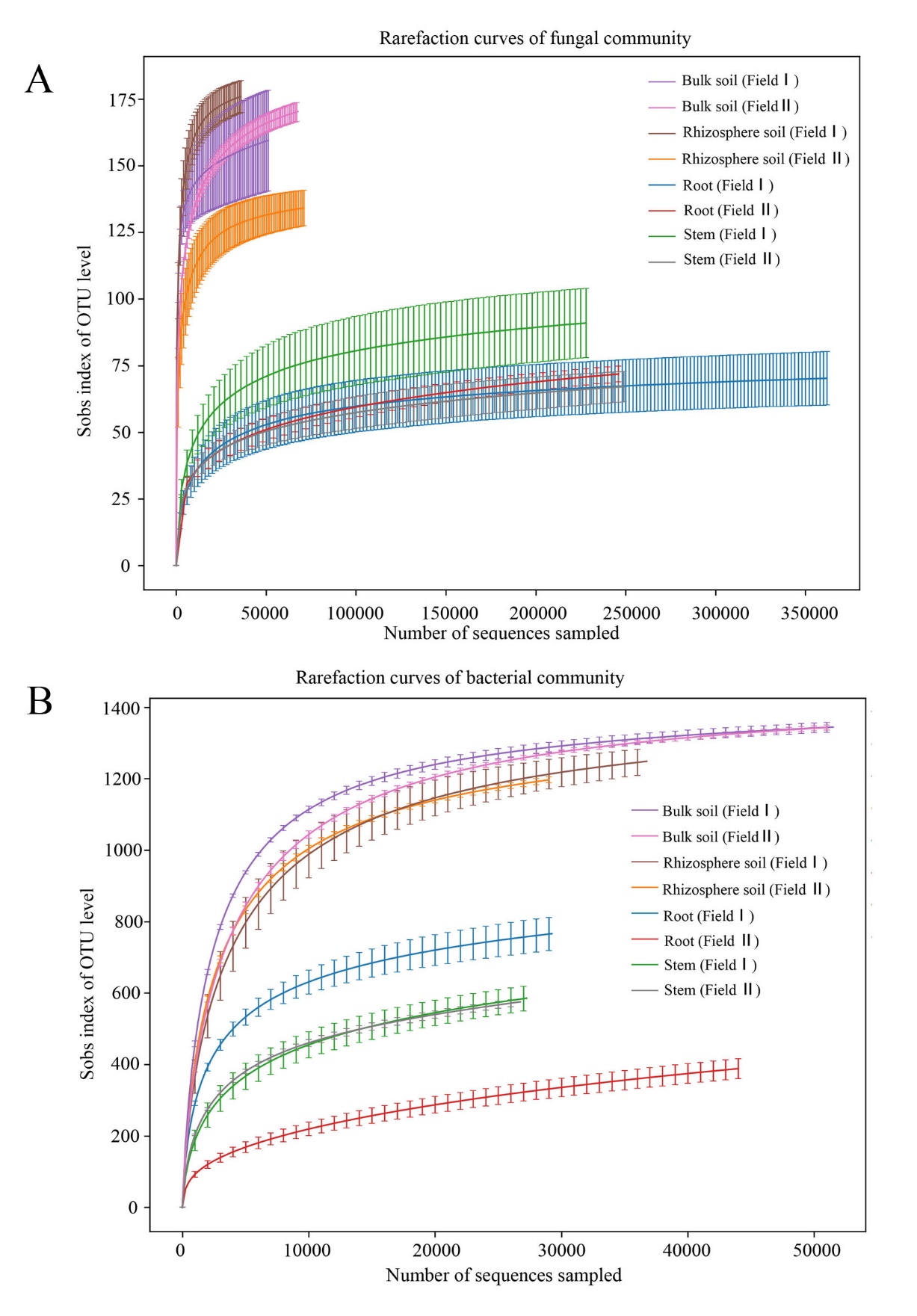


**Fig. S2.** Rarefaction curves of bulk soil, rhizosphere, root, and stem samples of *A. mongholicus* grown in the Field Ⅰ and Field Ⅱ. Curves representing the sequences of fungal OTUs are shown in (A) and those represent sequences for bacterial OTUs are shown in (B).


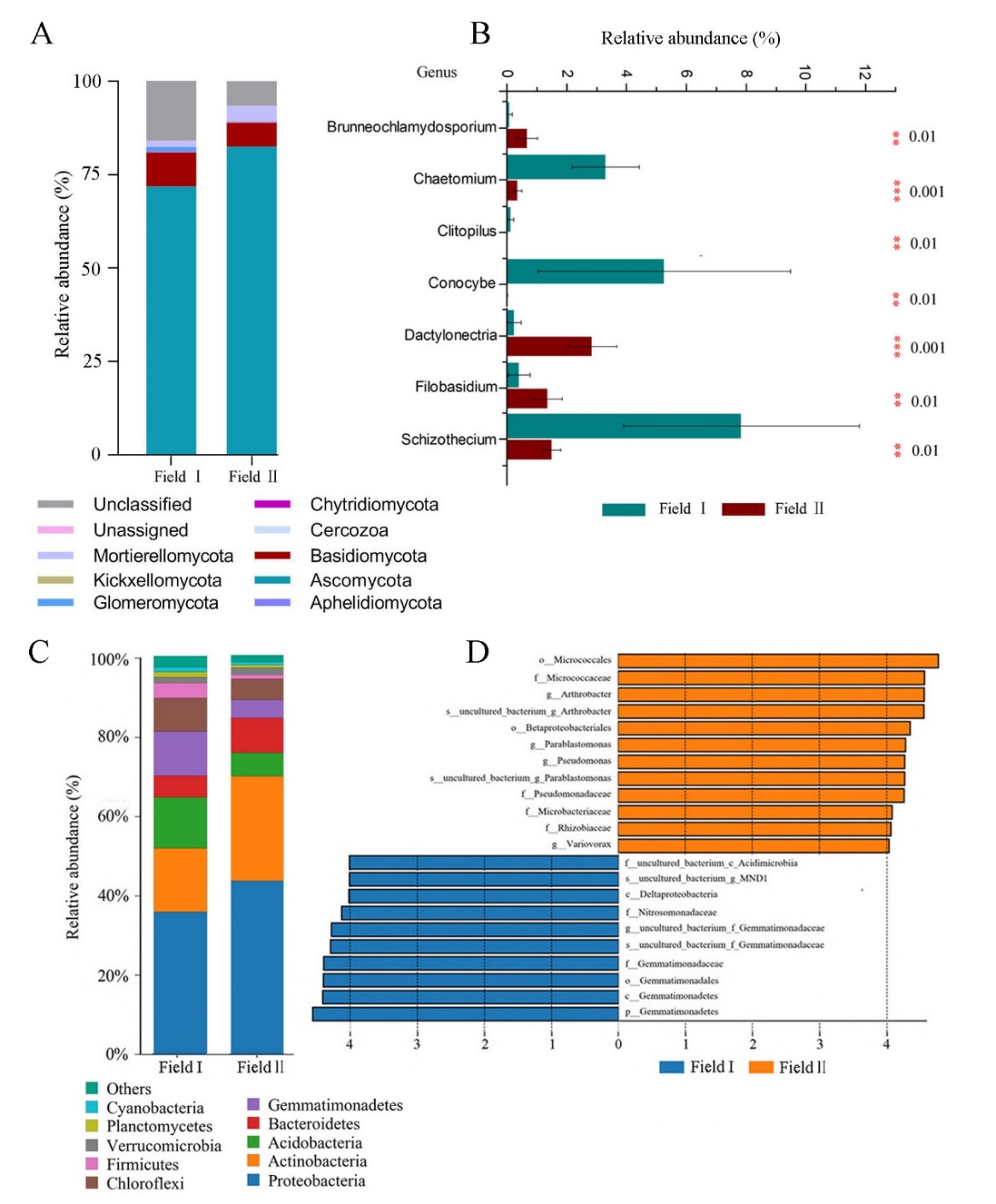


**Fig. S3.** Fungal and bacterial communities in the bulk soil of *A. mongholicus* grown in the FieldⅠand the FieldⅡ. (A) Taxonomic classification of fungal OTUs with relative abundance greater or equal to 2% grouped at the phylum level. The two bars represent the relative abundance of fungal phyla in the bulk soil of *A. mongholicus* grown in the FieldⅠand the FieldⅡ. (B) The relative abundance (%) of members of specific genera enriched in the bulk soil of *A. mongholicus* grown in the FieldⅠand the FieldⅡ and of the entire genus present here was significantly different (t-test: **p<0.01, ***p<0.001) between the rhizosphere of two fields. (C) Taxonomic classification of bacterial OTUs grouped at the phylum level. The two bars represent the relative abundance of bacterial phyla in the bulk soil of *A. mongholicus* grown in the FieldⅠand the FieldⅡ. Unclassified represents the species that has not been taxonomically annotated. (D) The linear discriminant analysis effect size (LEfSe) algorithm screens out the specific bacteria of the bulk soil of *A. mongholicus* grown in the FieldⅠ(blue) and the FieldⅡ(orange).


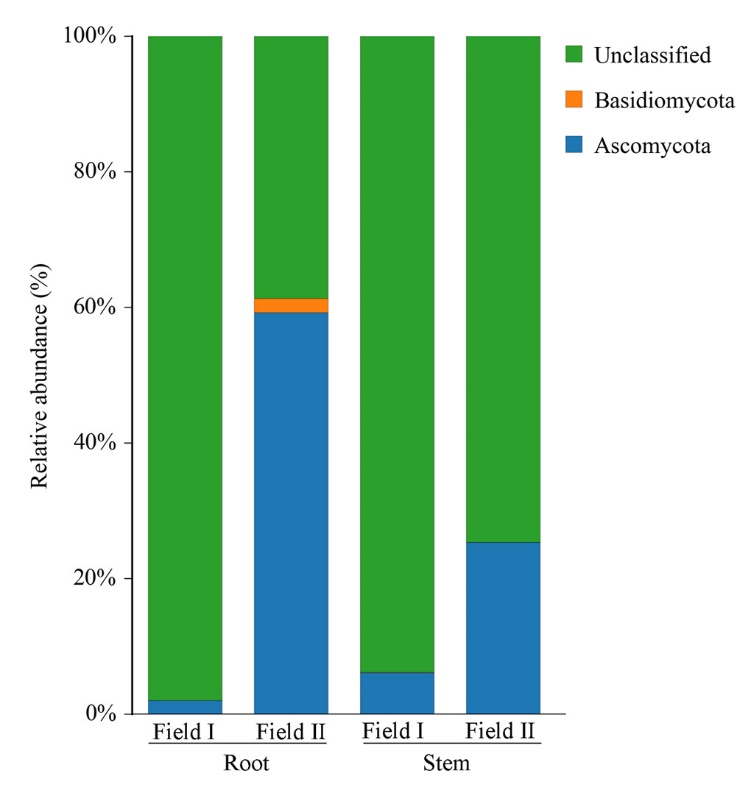


**Fig. S4.** Taxonomic classification of fungal OTUs with relative abundance greater or equal to 2% grouped at the phylum level. The four bars represent the relative abundance of fungal phyla in *A. mongholicus* root and stem grown in the FieldⅠand the FieldⅡ.


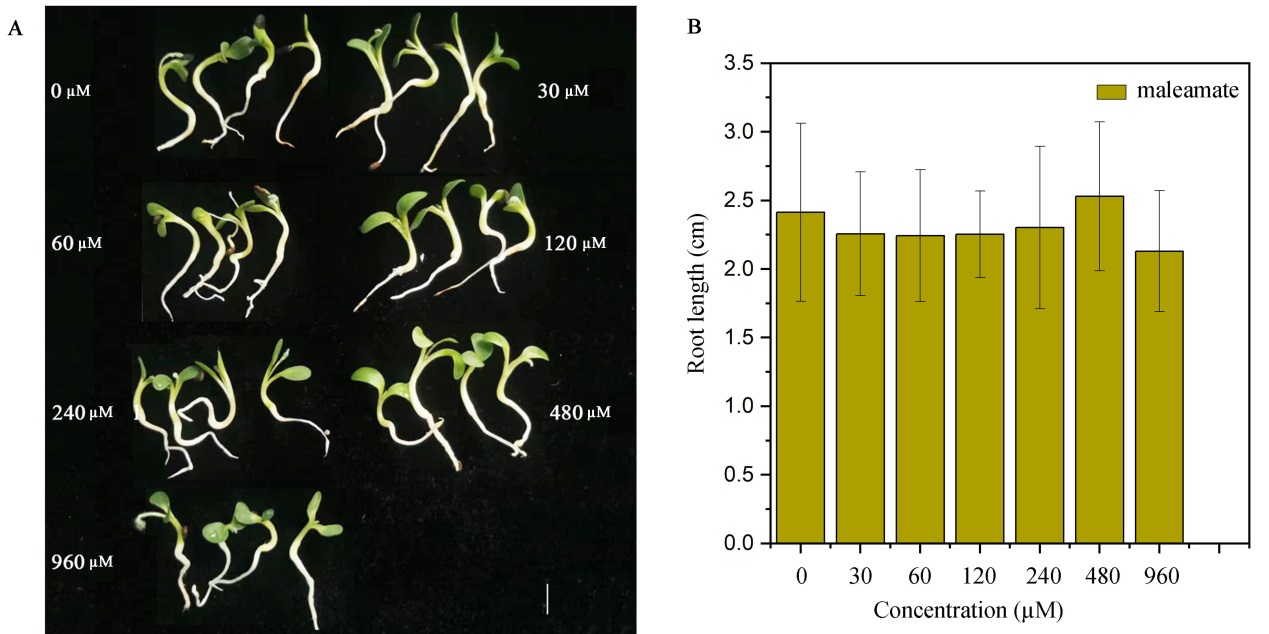


**Fig. S5.** The morphology and root length of 10-day-old seedlings of *A. mongholicus* treated with different concentrations of maleamate. Seedling morphology (A), and root length (B). Average values ± SD are shown (n=30) and Bar＝1 cm.


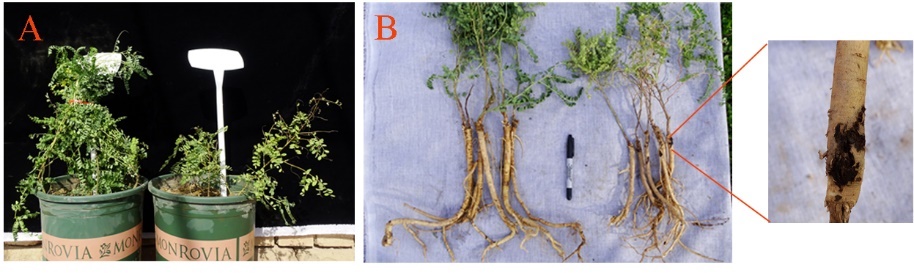


**Fig. S6.** Root symptoms of *A. mongholicus* with and without *F. oxysporum* infection. To test its pathogenicity, use a sterilized scalpel to make a 1 cm incision at the root about 3-5 cm from the junction of the root and stem of six healthy one-year old plants were pour-inoculated with a suspension of 3×10^5^ conidial/mL of HZ-F8 (50 mL) (A and B, left).The same number of plants were incised and poured with sterilized water as control (50 mL) (A and B, right). Two months after inoculation, root-rot symptoms were observed on root similar to those previously observed in the field (plants are enlarged).


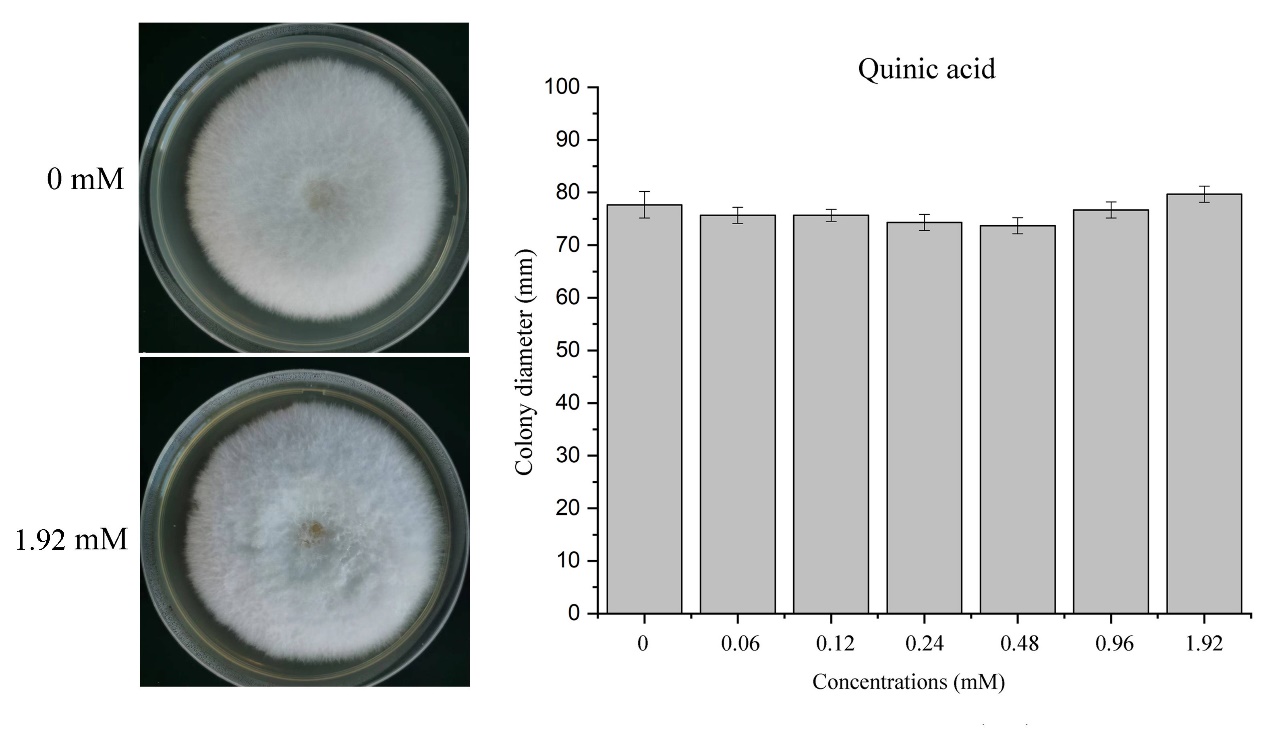


**Fig. S7.** Regulation of in vitro mycelial growth of *F. oxysporum* by quinic acid. (A) Colony formation of *F. oxysporum* isolate HZ-F8 on PDA media supplemented with different doses (0 and 1.92 mM) of quinic acid was investigated. (B) Relative mycelial growth regulated by quinic acid are shown as a percentage of fungal colonies on PDA, respectively.
